# Blueberries Reduce Palm Oil‐Induced Metabolic Endotoxemia in an In Vitro Human Intestinal‐Immune Cell Model

**DOI:** 10.1002/mnfr.70311

**Published:** 2025-11-11

**Authors:** Aina Casademont‐Roca, Monic M. M. Tomassen, Sander Kersten, Wilma T. Steegenga, Guido J. Hooiveld, Jurriaan J. Mes

**Affiliations:** ^1^ Division of Human Nutrition and Health Wageningen University & Research Wageningen The Netherlands; ^2^ Wageningen Food & Biobased Research Wageningen University & Research Wageningen The Netherlands; ^3^ Division of Nutritional Sciences Cornell University Ithaca New York USA

**Keywords:** berries, dietary fat, inflammation, intestine, LPS

## Abstract

Metabolic endotoxemia (ME), a dietary lipid‐induced increase in plasma LPS levels, is associated with cardiometabolic conditions. Accumulating evidence suggests an association between berry consumption and reduced endotoxemia. However, the underlying mechanisms remain unknown. This study examined the effects and potential mechanisms of blueberries, blackberries, and bananas on ME using an in vitro human intestinal‐immune cell model. Palm oil with LPS was added to intestinal Caco‐2 cells seeded on Transwell inserts, recapitulating dietary fat absorption. Higher levels of basolateral LPS were observed when Caco‐2 cells were cotreated with palm oil and LPS compared to control and LPS, supporting lipid‐induced LPS translocation. To examine the bioactivity of translocated LPS, THP1‐Lucia nuclear factor kappa B (NF‐κB) macrophages were exposed to basolateral conditioned media from Caco‐2 cells, and NF‐κB activation was assessed. Basolateral conditioned medium from Caco‐2 cells cotreated with digested palm oil and LPS induced higher macrophage NF‐κB activation compared to only palm oil. Interestingly, fruits reduced the palm oil + LPS‐mediated NF‐κB activation in macrophages. Transcriptomic and protein‐level analyses suggest berries modulate the lipid‐induced LPS translocation, likely via clathrin‐mediated transcytosis with a minor chylomicron‐mediated contribution. The anti‐inflammatory effects of berry‐rich diets may be mediated by preventing ME.

## Graphical abstract

Digested palm oil and LPS treatment on intestinal Caco‐2 cells results in palm oil‐induced LPS translocation across the intestinal cell monolayer and nuclear factor kappa B (NF‐κB) activation of basolateral macrophages. Interestingly, digested whole berries reduce the palm oil + LPS‐mediated NF‐κB activation in macrophages. These findings suggest an anti‐inflammatory role of berries in the context of metabolic endotoxemia.

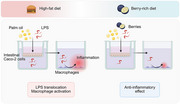

**Abbreviations**
ANOVA: analysis of variance
AP3B1: adaptor related protein complex 3 subunit beta 1
APOB: apolipoprotein B
BB: blueberry
BK: blackberry
BN: banana
CFR: Code of Federal Regulation
CHMP2B: charged multivesicular body protein 2B
CLHC1: clathrin heavy chain linker domain containing 1
cpm: counts per million reads
Ctrl: control
ZDHHC: zinc finger aspartate‐histidine‐histidine‐cysteine
DNM1L: dynamin 1‐like
EEA1: early endosome antigen 1
EV: extracellular vesicle
FABP2: fatty acid binding protein 2
FCHO2: FCH and mu domain containing endocytic adaptor 2
FCS: fetal calf serum
FDR: false discovery rate
Fisher's LSD: Fisher's Least Significant Difference
GOLGA4: golgin A4
GOLGA8A: golgin A8 family member A
GOLGA8B: golgin A8 family member B
HF: high‐fat
HFHS: high‐fat/high‐sucrose
HSP90AA1: heat shock protein 90 kDa alpha family class A member 1
HSP90B1: heat shock protein 90 kDa beta family member 1
ITSN1: intersectin 1
ITSN2: intersectin 2
KEGG: Kyoto Encyclopedia of Genes and Genomes
LY: Lucifer Yellow
ME: metabolic endotoxemia
MTTP: microsomal triglyceride transfer protein
MVE: multivesicular endosome
Mw: molecular weight
MYO6: myosin VI
NF‐κB: nuclear factor kappa‐light‐chain‐enhancer of activated B cells
P/S: penicillin/streptomycin
PCA: principal component analysis
pKFlex: Pyros Kinetix Flex tube reader
PMA: phorbol 12‐msyristate 13‐acetate
RAB27B: RAB27B, member RAS oncogene family
RAB9B: RAB9B, member RAS oncogene family
RLU: relative luminescence unit
RPMI medium: Roswell Park Memorial Institute medium
STON1: stonin 1
TEER: transepithelial electrical resistance
TLR4: Toll‐like receptor 4
TX‐100: Triton‐X 100
WASHC4: WASH complex subunit 4
WST‐1: water‐soluble tetrazolium salt‐1

## 1 Introduction

Metabolic endotoxemia (ME) is characterized by an increase in circulating LPS from enteric bacteria following consumption of a high‐fat (HF) meal [1]. Cani et al. were the first to demonstrate that serum LPS levels after a 4‐week HF diet exposure were comparable to those in mice continuously infused with LPS for 4 weeks [1]. Indeed, plasma LPS levels have been observed to increase after a fat challenge in clinical [2, 3, 4, 5, 6], animal [1, 7, 8, 9, 10], and in vitro [7, 11] studies. In pre‐clinical stages of ME, it has been suggested that LPS may translocate across the intestinal epithelium via transcellular mechanisms mediated by chylomicrons [7, 11, 12] and transcytosis [8, 13, 14]. However, in the context of persistent ME, increased intestinal permeability might occur, and LPS may cross the intestinal barrier in a paracellular manner [15]. LPS in plasma activates the immune system and contributes to inflammatory diseases such as cardiometabolic and neurodegenerative conditions [16, 17, 18]. Hence, identifying effective dietary strategies to prevent ME is essential for alleviating this burden.

Although research is limited and primarily based on pre‐clinical studies, growing evidence suggests a link between the consumption of berries or berry‐derived compounds and reduced endotoxemia. Anhê et al. demonstrated that different berry extracts (e.g., blueberry [BB], blackberry [BK], and lingonberry) could reduce circulating LPS levels in mice fed with a high‐fat/high‐sucrose (HFHS) diet [19]. Likewise, a pre‐clinical study by Nunes et al. showed a trend toward reduced plasma LPS levels in a hypercaloric diet‐induced prediabetic rat model supplemented with BB juice [20]. In addition to reducing plasma LPS, supplementation of berries (i.e., lingonberries) on HF‐fed mice significantly decreased circulating LPS binding protein [21], a marker of LPS exposure in plasma with a half‐life longer than LPS [22]. Similarly, epidemiological data point to an association between berry consumption and reduced systemic endotoxemia in 688 individuals with Type 1 diabetes [23].

Given the limited understanding of causality and the molecular mechanisms underlying the association between berry intake and reduced endotoxemia, this study investigated the effects and potential mechanisms of in vitro digested whole, BBs, BKs, and bananas (BNs) on intestinal ME by using a human intestinal Caco‐2/THP1‐Lucia NF‐κB macrophage cell model.

## 2 Experimental Section

### 2.1 Cell Culture and Differentiation Methods

Caco‐2 cells (HTB‐37, ATCC) were cultured in Dulbecco's Modified Eagle Medium (DMEM) (Corning) supplemented with 10% fetal calf serum (FCS) (Biowest) and 1% penicillin/streptomycin (P/S) until 80%–90% confluence at 37°C/5% CO_2_. Cells were used in passage numbers between 30 and 40. Caco‐2 cells were differentiated into small intestine‐like epithelial cells as previously described [11]. Briefly, cells were seeded in 24‐well, translucent, ThinCert cell culture inserts with 0.4 µm pores (Greiner‐Bio One), in a volume of 150 µL with a seeding density of 225 000 cells/mL and 700 µL DMEM supplemented with 10 % FCS and 1 % P/S in the basolateral compartment. Caco‐2 cells were differentiated during 21 days at 37°C/5% CO_2_ and were apically deprived of FCS from Day 14 to 21. Apical and basolateral media were refreshed three times a week and 1 day before exposure treatment.

The human THP1‐Lucia NF‐κB monocyte cells (thpl‐nfkb, InvivoGen), derived from the human THP1 monocyte cell line by stable integration of an nuclear factor kappa‐light‐chain‐enhancer of activated B cells (NF‐κB) inducible luciferase reporter construct, enabled monitoring of NF‐κB activation by assessing the activity of secreted Lucia luciferase. THP1‐Lucia NF‐κB monocyte cells were cultured in Roswell Park Memorial Institute (RPMI) 1640 Medium (ThermoFisher) supplemented with 10% FCS (Biowest), 1% P/S, and 2 mM GlutaMAX (ThermoFisher) at 37°C/5% CO_2_. THP1‐Lucia NF‐κB monocyte cells were used until Passage 25. To maintain selection pressure, every other passage was supplemented with 100 µg/mL Zeocin (InvivoGen). Differentiation of THP1 monocyte cells to macrophages was performed as previously described [24, 25]. THP1‐Lucia NF‐κB monocyte cells were plated in a seeding density of 315 789 cells/cm^2^ in 24‐well or 48‐well plates, and differentiated into macrophage‐like cells after treatment with 50 ng/mL phorbol 12‐msyristate 13‐acetate (PMA) (Sigma) for 72 h at 37°C/5% CO_2_. PMA was washed off, and cells were rested in RPMI without phenol red supplemented with 10% FCS, 1% P/S, and 2 mM GlutaMAX for 48 h at 37°C/5% CO_2_. After incubation, macrophage‐like cells were exposed to the treatments.

### 2.2 In Vitro Gastrointestinal Digestions: Fruits and Palm Oil

In vitro gastrointestinal digestion of fruits and palm oil was performed as described by Tomassen et al. [11], who developed a modified INFOGEST [26] protocol optimized for Caco‐2 cell exposure studies. BBs (*Vaccinium corymbosum*), BKs (*Rubus fruticosus*), and BNs (*Musa acuminata*) were purchased from the two local Dutch supermarkets Jumbo and Albert Heijn (Wageningen, the Netherlands). Two separate batches of each fruit were combined to minimize batch‐to‐batch variation. The fresh fruits were then freeze‐dried and finely ground into powder using a coffee grinder. Palm oil was purchased from Research Diet Services B.V. (for a more detailed composition, see Supporting Information Table S1). Briefly, the fruit powders and palm oil were diluted to a concentration of 0.2 g/mL using a solution containing 140 mM NaCl and 5 mM KCl, making a total volume of 20 mL. The fruit mixtures were gently vortexed to a homogeneous solution, while the palm oil mixture was gently vortexed and heated at 56°C for 15 min. To mimic the gastric digestion phase, the pH of the samples was adjusted to 2 using 1 M HCl. A pepsin solution (1092 U/mL dissolved in 0.1 M HCl) was then added, and the samples were incubated at 37°C for 1 h under gentle agitation. To simulate intestinal digestion, the pH was adjusted to 5.8 using 1 M NaHCO_3_, followed by the addition of 4 mg/mL pancreatin (6.84 U/mg trypsin activity), 5.9 U/mL α‐chymotrypsin (65.62 U/mg), 1 mg/mL lipase (all from porcine), and bile salts (94.6 mg/mL sodium taurocholate and 83 mg/mL sodium glycodeoxycholate) dissolved in 0.1 M NaHCO_3_. The pH was then adjusted to 6.5 with 1 M NaHCO_3_, and the samples were incubated at 37°C for 2 h under gentle agitation. After incubation, the pH was further adjusted to 7.5 with 1 M NaHCO_3_, and the digested volume was increased to 30 mL with 140 mM NaCl and 5 mM KCl. Samples were centrifuged at 3023 × *g* for 30 min at 4°C, after which the supernatants were snap‐frozen with liquid nitrogen and stored at −80°C until further use. As a negative control, digested controls were prepared that included all buffers and enzymes but excluded the fruit powders and palm oil.

### 2.3 In Vitro Treatments

#### 2.3.1 Palm Oil‐Induced LPS Translocation Experiments

Differentiated Caco‐2 cells were apically exposed to digested control or palm oil (1:50 diluted) without or with 0.5 mg/mL LPS from *Escherichia coli* O111:B4 (Sigma) for 24 h at 37°C/5% CO_2_. All investigational exposures were performed in DMEM without phenol red (ThermoFisher) and FCS (as it interferes with LPS detection), supplemented with 1 mM sodium pyruvate (ThermoFisher) and 1% P/S. In the basolateral compartment, phenol red‐free Roswell Park Memorial Institute medium (RPMI medium, ThermoFisher) supplemented with 2 mM GlutaMAX (ThermoFisher) and 1% P/S was added. A final volume of 150 and 700 µL was added to the apical and basolateral compartments, respectively. After a 24 h incubation at 37°C/5% CO_2_, the basolateral conditioned medium was used for further analysis. Experiments were conducted in three technical and three biological replicates.

#### 2.3.2 Blueberry, Blackberry, and Banana Exposure Experiments

Differentiated Caco‐2 cells were apically exposed to digested palm oil (1:50 diluted) and digested control, BBs, BKs, or BNs (1:4 diluted), without or with LPS (0.5 mg/mL). All apical investigational exposures were performed in DMEM without phenol red (ThermoFisher) and FCS, supplemented with 1 mM sodium pyruvate (ThermoFisher) and 1% P/S. In the basolateral compartment, phenol red‐free RPMI medium (ThermoFisher) supplemented with 2 mM GlutaMAX (ThermoFisher), 10% FCS, and 1% P/S was added. A final volume of 150 and 700 µL was added to the apical and basolateral compartments, respectively. After a 24 h incubation at 37°C/5% CO_2_, the basolateral conditioned mediums were collected and stored at −80°C until further use. Caco‐2 cell basolateral conditioned mediums were thawed and used for different purposes: (A) 450 µL was added to THP1‐Lucia NF‐κB macrophages (in a 24‐well plate) for 24 h at 37°C/5% CO_2_, and (B) assessment of chylomicron secretion by Caco‐2 cells. Experiments were conducted in three technical and three biological replicates.

The amounts of fruit used in this study closely reflect physiological conditions. Specifically, the tested amounts correspond to the consumption of approximately 350 g fresh berries or 150 g BN (1 medium BN), which are in line with the dietary intake of fruits and vegetables as recommended by the European Food Safety Authority (EFSA) [27, 28].

#### 2.3.3 Lomitapide Blockage Experiments

To examine whether palm oil‐induced LPS translocation was chylomicron‐mediated, the chylomicron inhibitor Lomitapide [29] was added. Differentiated Caco‐2 cells were apically exposed to palm oil and Lomitapide (1 µM) or DMSO (0.1%, which served as vehicle), without or with LPS (0.5 mg/mL), for 24 h at 37°C/5% CO_2_. All apical investigational exposures were performed in DMEM without phenol red (ThermoFisher) and FCS, supplemented with 1 mM sodium pyruvate (ThermoFisher) and 1% P/S. In the basolateral compartment, phenol red‐free RPMI medium (ThermoFisher) without FCS, supplemented with 2 mM GlutaMAX (ThermoFisher), and 1% P/S was added. A final volume of 150 and 700 µL was added in the apical and basolateral compartments, respectively. After a 24 h incubation at 37°C/5% CO_2_, the basolateral conditioned mediums were collected and stored at −80°C until further use. Caco‐2 cell basolateral conditioned mediums were thawed and used for different purposes: (A) a final volume of 300 µL, supplemented with fresh 10% FCS, was exposed to THP1‐Lucia NF‐κB macrophage cells (in 48‐well plate) for 24 h at 37°C/5% CO_2_, and (B) assessment of chylomicron secretion by Caco‐2 cells. Experiments were conducted in three technical and three biological replicates.

### 2.4 Trans‐ and Paracellular Epithelial Permeability Assays

To monitor the integrity of the Caco‐2 cell monolayer, transepithelial electrical resistance (TEER) was measured using an EVOM2 Volt/Ohm meter using STX2 electrodes (World Precision Instruments, Sarasota, USA) on a heating plate maintained at 37°C. TEER values after incubation with the investigational exposures were expressed as a percentage of the TEER values measured just before adding the compounds. Differentiated Caco‐2 monolayers were considered of acceptable quality when TEER values were higher than 700 Ω [30]. To examine the paracellular permeability of particles with a molecular weight (Mw) higher than 0.4 kDa, the Lucifer yellow (LY) assay was performed as follows. After TEER measurements, culture inserts were washed with PBS and transferred to a new 24‐well plate. LY CH dilithium salt of 0.4 kDa (Sigma) was dissolved in DMEM without phenol red (Thermofisher) supplemented with 1 mM sodium pyruvate (ThermoFisher) and 1% P/S to 1 mg/mL, and 100 µL was added to the apical compartment. In the basolateral compartment, 700 µL of phenol red‐free RPMI supplemented with 2 mM GlutaMAX (ThermoFisher) and 1% P/S was added, and then the plate was incubated for 3 h at 37°C/5% CO_2_. After incubation, 100 µL of the basolateral compartment was collected, and fluorescence was measured at 425/515 nm (excitation/emission). For 100% LY paracellular permeability, empty cell culture inserts were used.

### 2.5 Detection of LPS

#### 2.5.1 Pyros Kinetix Flex

LPS was measured in basolateral conditioned media from Caco‐2 cells with a chromogenic assay from Nodia (Associates of Cape Cod, Inc.). The assay was performed in a Pyros Kinetix Flex tube reader (pKFlex) (Nodia, Cape Cod, Inc.). Pyrochrome lysate was reconstituted with 3.4 mL Pyrochrome reconstitution buffer (C1500‐5), and for the calibration curve, Limulus amebocyte lysate control standard with an endotoxin concentration of 0.5 µg/vial (CSEE0005‐1) was used. The samples were diluted with LPS‐free Milli‐Q water and incubated at 70°C for 15 min. After cooling for 1 h at 4°C, the samples were placed at room temperature for 15 min and then immediately measured using the pKFlex. Thus, 200 µL of treated sample or standard was added to a pKFlex glass tube, followed by the addition of 50 µL of Pyrochrome. The mixture was briefly stirred and then placed in the pKFlex for measurement. The endotoxin concentration was calculated using the Pyros eXpress software, compliant with 21 Code of Federal Regulations (CFRs) Part 11.

#### 2.5.2 EndoZyme

To quantify the presence of LPS in digested fruits, palm oil, and control preparations, the EndoZyme assay (Hyglos GmbH, Bernried am Starnberger See, Germany) was used following the manufacturer's instructions and including positive endotoxin recovery controls.

### 2.6 NF‐κB Pathway Detection With THP1‐Lucia NF‐κB Reporter Cell Line

**FIGURE 1:**
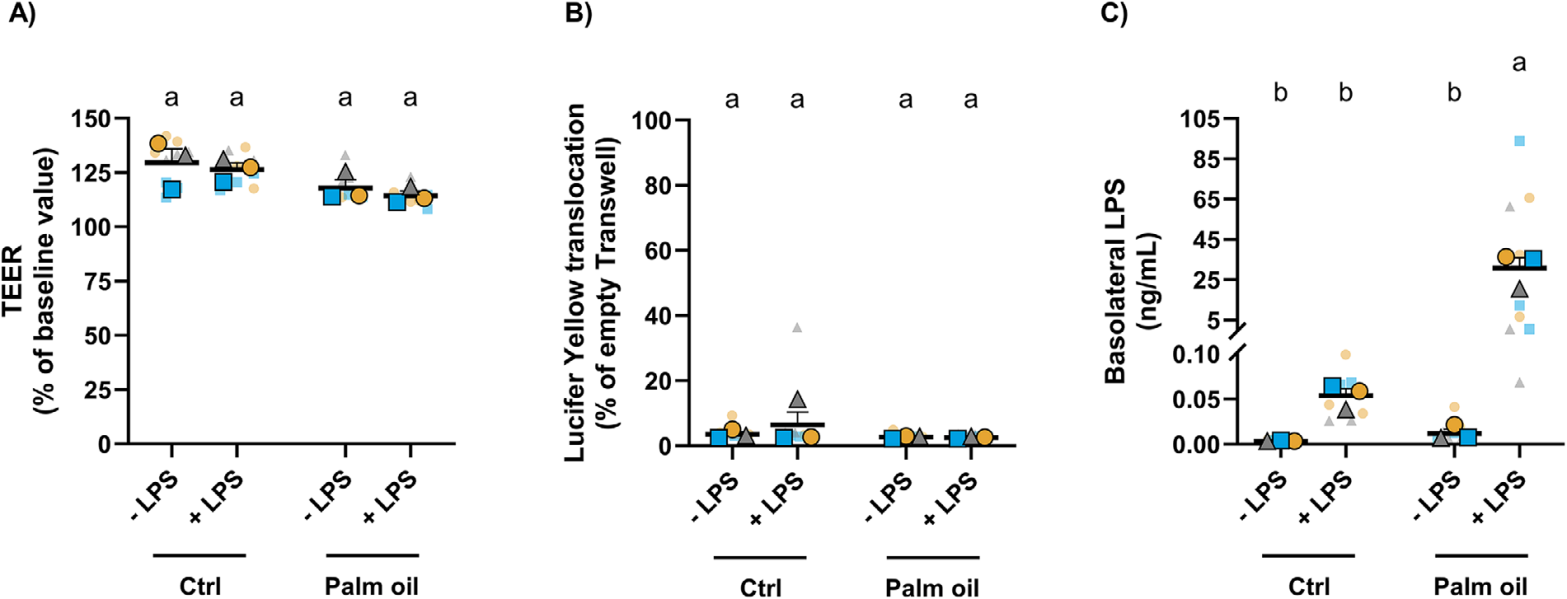
Digested palm oil facilitates LPS translocation across a confluent Caco‐2 cell monolayer. (A) TEER values. (B) Basolateral levels of LY. (C) Basolateral LPS levels. Statistical differences were determined using a random block two‐way ANOVA to account for differences due to Caco‐2 cell passage number (A, C) or an ordinary two‐way ANOVA (B). Post hoc pairwise comparisons were performed using Fisher's LSD test. Different letters (a–b) indicate a significant difference between experimental groups (*p* ≤ 0.05), as determined by ANOVA. Data are presented as the mean ± SEM from three technical and three biological replicates. ANOVA, analysis of variance; Fisher's LSD, Fisher's Least Significant difference; LY, Lucifer Yellow; SEM, standard error of the mean; TEER, transepithelial electrical resistance.

To assess activation of the NF‐κB signaling pathway in THP1‐Lucia NF‐κB macrophages (thpl‐nfkb, InvivoGen), the activity of secreted Lucia luciferase in the macrophage‐conditioned medium was measured. Thus, 10 µL of macrophage‐conditioned medium was transferred to a white opaque 96‐well plate (Greiner Bio‐One), and luminescence was measured using a VANTAstar plate reader (Isogen) following automated injection of 50 µL of the luciferase detection QUANTI‐Luc4 reagent (rep‐qlc4lg1, InvivoGen). Luminescence was measured in macrophage‐conditioned medium and in cell‐free medium (phenol red‐free RPMI medium, supplemented with 2 mM GlutaMAX, 10% FCS, and 1% P/S), which served as the background negative control medium. Cell‐free medium luminescence was subtracted from all sample readings to obtain corrected values, which were then expressed in relative luminescence units (RLUs). THP1‐Lucia NF‐κB macrophages stimulated with LPS (10 or 30 ng/mL) were used as a positive control for NF‐κB activation, while macrophages cultured in RPMI medium alone served as a negative control. The 10 ng/mL LPS concentration was initially selected since it was recommended by the manufacturer (InvivoGen), while the 30 ng/mL LPS concentration was selected based on the measured amount of LPS translocated to the basolateral compartment of the intestinal Caco‐2 cell LPS translocation model (Figure 1C).

### 2.7 WST‐1 Assay

Cytotoxicity was assessed by the water‐soluble tetrazolium salt‐1 (WST‐1) assay (Merck) following the manufacturer's instructions. Briefly, absorbance of the formazan dye produced by metabolically active cells was measured at 450 nm (reference wavelength of 690 nm) using a VANTAstar plate reader (Isogen). Absorbance was measured in treated samples and in cell‐free medium (phenol red‐free RPMI medium, supplemented with 2 mM GlutaMAX, 10% FCS, and 1% P/S), which served as the background negative control medium. Cell‐free medium absorbance was subtracted from all sample readings to obtain corrected values, which were then expressed as a percentage relative to untreated macrophages (negative control). As a positive control for maximal cytotoxicity, macrophages were treated with 1% Triton‐X 100 (TX‐100).

### 2.8 Detection of the Chylomicron Marker APOB

Basolateral conditioned medium from treated Caco‐2 cells was collected and used for the assessment of apolipoprotein B (APOB). Levels of APOB were measured with the human APOB ELISA kit (ABIN612664, Antibodies Online) following the manufacturer's instructions.

### 2.9 Fatty Acid Profile Analysis of Palm Oil

Fatty acid profile was determined by standard preparation of methyl esters from extracted fat, and individual fatty acids were quantified in relative percentages by gas chromatography with downstream flame ionization detector as outsourced at certified analytic laboratory Eurofins (Eurofins Food Testing, Heerenveen, the Netherlands).

### 2.10 RNA Isolation and Sequencing

RNA was extracted from cells by TRIzol reagent (Life Technologies, the Netherlands) and subsequently purified with RNeasy spin columns (Qiagen, the Netherlands).

RNA sequencing was performed on exposed Caco‐2 cells (pooled from three technical replicates, with three independent biological replicates per group). RNA integrity was measured by Agilent 2100 Bioanalyzer with RNA 6000 microchips (Agilent Technologies, Santa Clara, CA). Library construction and RNA sequencing runs on the BGISEQ‐500 platform were conducted at Beijing Genomics Institute (BGI, Hong Kong). At BGI, Genomic DNA was removed with two digestions using Amplification grade DNAse I (Invitrogen, USA). The RNA was sheared and reverse transcribed using random primers to obtain cDNA, which was used for library construction. The library quality was determined using a Bioanalyzer 2100. Thereafter, the library was used for 100 bp paired‐end sequencing at 20 million reads per sample on the BGISEQ‐500 sequencing platform (BGI). All the generated raw sequencing reads were filtered by removing reads with adaptors, reads with more than 10% of unknown bases, and low‐quality reads. Clean reads were then obtained and stored in FASTQ format.

### 2.11 Processing of RNA Sequencing Reads

The RNA sequencing reads were used to quantify transcript abundances. The tool *salmon* (version 1.10.0) [31] was used to map the reads to the GRCh38.p14 human genome assembly‐based transcriptome sequences as annotated by the GENCODE consortium (release 46) [32]. The obtained transcript abundance estimates and lengths were imported in R using the package *tximeta* (version 1.22.1) [33], scaled by average transcript length and library size, and summarized at the gene level. Differential gene expression was determined using the package *limma* (version 3.60.6) [34, 35] utilizing the obtained scaled gene‐level counts. Briefly, before statistical analyses, nonspecific filtering of the count table was performed to increase detection power [36] based on the requirement that a gene should be detected with at least 10 counts, for at least 6 libraries, across all 24 libraries. This cutoff thus corresponds to ∼0.5 counts per million reads (cpm) mapped. Differences in library size were adjusted by the trimmed mean of *M* values normalization method [37], implemented in the package *edgeR* (version 4.2.1) [38]. Before subjecting the RNA‐seq data to linear modeling, the count data were *voom*‐transformed [39]. In short, counts were transformed to Log2 (cpm) values, and the mean–variance relationship in the data was estimated. This relationship was used to compute a precision weight for each observation. The weights were then used in the linear modeling process to adjust for heteroscedasticity. Differentially expressed genes were identified by general linear modeling that incorporates empirical Bayesian estimators to guide prior parameter specification, as implemented in the package *limma* [34, 35]. To control for multiple significance testing, a false discovery rate (FDR) was calculated according to the Benjamini–Hochberg procedure [40].

### 2.12 Data Analyses

Statistical analyses were conducted using GraphPad Prism version 10.2.2 (San Diego, CA, USA). Normality and homogeneity of variance were assumed, unless stated otherwise. Comparisons including three or more groups with one independent variable were analyzed using one‐way analysis of variance (ANOVA), while comparisons with two independent variables were assessed using two‐way ANOVA. Where applicable, random block one‐ or two‐way ANOVA was conducted to account for differences due to Caco‐2 cell passage number [41, 42]. Post hoc pairwise comparisons were performed using Fisher's Least Significant Difference (Fisher's LSD) test. Different letters (a–c) indicate a significant difference between experimental groups (*p* ≤ 0.05), as determined by ANOVA, unless stated otherwise. Data are presented as the mean ± standard error of the mean (SEM) from three technical and three biological replicates, unless stated otherwise. For RNA‐seq data, genes were defined as significantly changed based on FDR ≤ 0.05.

## 3 Results

### 3.1 Digested Palm Oil Facilitates LPS Translocation Across a Confluent Human Intestinal Caco‐2 Cell Monolayer

The first step of this study was to establish an in vitro human intestinal cell model in the context of ME. In vitro digested palm oil or its respective digested control was added without or with LPS to differentiated Caco‐2 cells for 24 h. TEER values (expressed as % of baseline value) were used as the first indication of Caco‐2 cell monolayer integrity, and treatment exposures were maintained above 100% (Figure 1A). The same Caco‐2 cell culture inserts were subjected to the 0.4 kDa LY paracellular permeability assay. All assessed treatment exposures showed that, on average, 3.8 ± 2.0% of LY translocated to the basolateral compartment, when compared to empty inserts representing 100% LY translocation (Figure 1B). Subsequent analysis of LPS transport across the confluent Caco‐2 cell monolayer revealed significantly higher basolateral LPS concentrations in cells coincubated with palm oil and LPS compared to cells coincubated with control and LPS (Figure 1C). It is worth noting that basolateral LPS levels showed an increasing trend in Caco‐2 cells that had been coincubated with digested control and LPS compared to the digested control alone. However, this difference did not reach statistical significance. To confirm the absence of significant amounts of LPS in the digested control and palm oil samples, LPS levels were measured and found to be negligible compared to the 0.5 mg/mL LPS added in the final cell model (Supporting Information Table S2). Based on these findings, exposure to palm oil and LPS was selected as a positive control for ME for the remainder of the study.

### 3.2 Digested Blueberries, Blackberries, and Bananas Reduce NF‐κB Signaling Pathway in Macrophages During Palm Oil‐Induced Metabolic Endotoxemia

**FIGURE 2:**
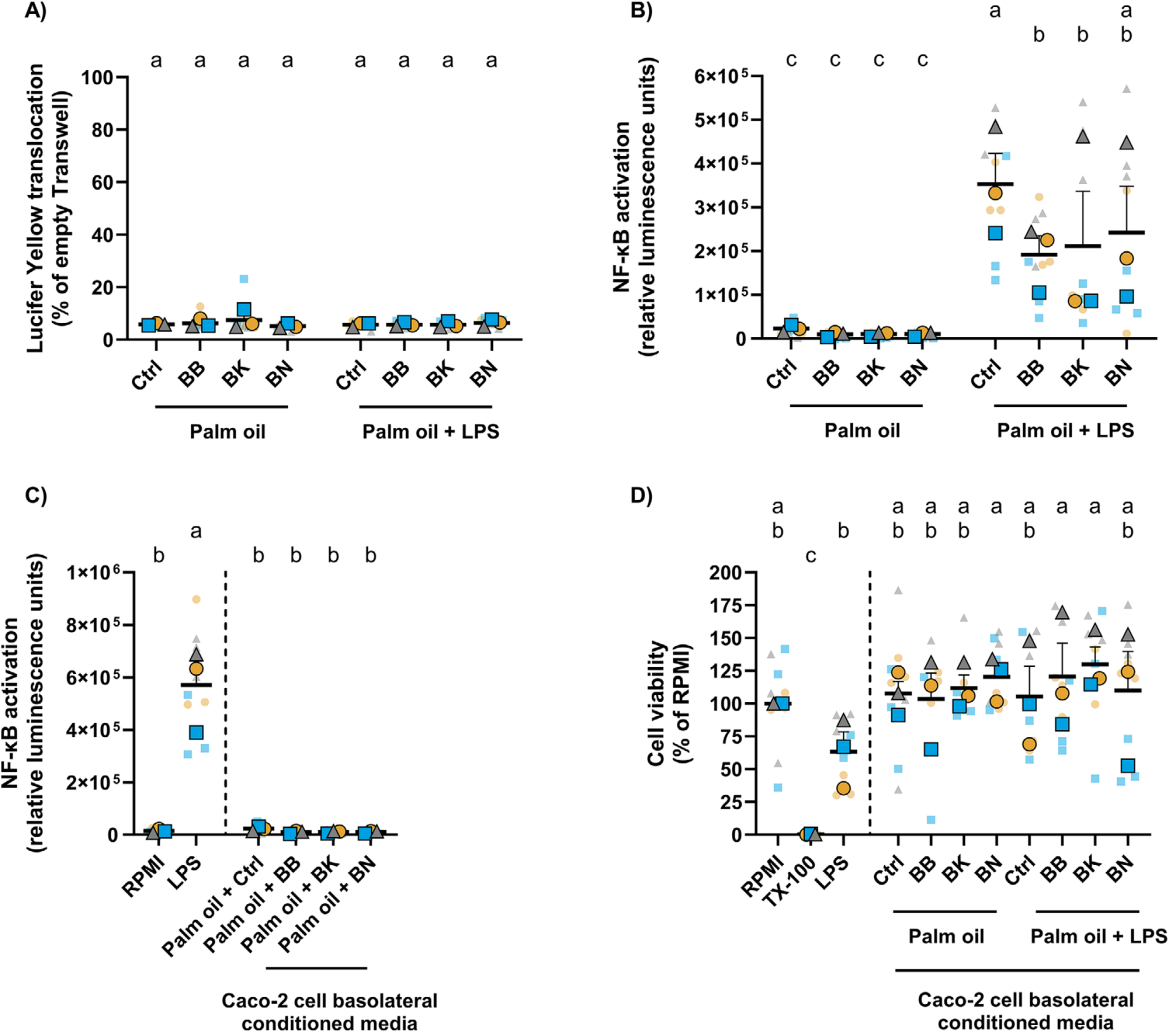
Digested fruits reduce palm oil + LPS‐mediated NF‐κB activation in THP1‐Lucia NF‐κB macrophages. (A) Effects of digested fruits (BB, BK, or BN) or control alongside palm oil, without or with LPS, on Caco‐2 cell permeability examined by the LY assay. (B) NF‐κB activation in macrophages after 24 h incubation with basolateral conditioned media from Caco‐2 cells that had been cotreated with digested fruits or control alongside palm oil, without or with LPS for 24 h. (C) NF‐κB activation in macrophages after exposure to RPMI, LPS (10 ng/mL), or basolateral conditioned media from Caco‐2 cells that had been cotreated with palm oil and fruits or control. (D) Viability of macrophages treated with RPMI, TX‐100 (1%), LPS (10 ng/mL), or basolateral conditioned media from Caco‐2 cells that had been cotreated with digested fruits or control alongside palm oil, without or with LPS. Statistical differences were determined using an ordinary two‐way (A) or one‐way (D) ANOVA; or random block two‐way (B) or one‐way (C) ANOVA to account for differences due to Caco‐2 cell passage number. Post hoc pairwise comparisons were performed using Fisher's LSD test. Different letters (a–c) indicate a significant difference between experimental groups (*p* ≤ 0.05), as determined by ANOVA. Data are presented as the mean ± SEM from three technical and three biological replicates. ANOVA, analysis of variance; BB, blueberry; BK, blackberry; BN, banana; Fisher's LSD, Fisher's Least Significant Difference; LY, Lucifer Yellow; NF‐κB, nuclear factor kappa B; RPMI, Roswell Park Memorial Institute; SEM, standard error of the mean; TX‐100, Triton‐X 100.

The effects of digested fruits on the intestinal ME cell model were next evaluated. To this end, digested BBs, BKs, BNs, or their respective control were coincubated alongside the digested palm oil, without or with LPS, on differentiated Caco‐2 cells for 24 h. No significant differences in Caco‐2 cell monolayer permeability were found with the LY assay when comparing digested fruits to the digested control (Figure 2A). Subsequently, basolateral Caco‐2 cell‐conditioned media was added to THP1‐Lucia NF‐κB macrophages for 24 h. NF‐κB activation in macrophages was used to assess the bioactivity of potential palm oil‐induced translocated LPS. Basolateral conditioned medium from Caco‐2 cells that had been cotreated with digested palm oil and LPS induced significantly higher activation of the NF‐κB pathway in macrophages compared to basolateral conditioned medium from Caco‐2 cells treated with only digested palm oil. Interestingly, BBs significantly reduced the palm oil + LPS‐mediated NF‐κB activation in macrophages (*p* = 0.0120), as did BKs (*p* = 0.0205), while a trend toward a reduction was observed for BNs (*p* = 0.0509) (Figure 2B). Average percentage reductions in macrophage NF‐κB activation for each fruit treatment, relative to the positive control (palm oil + LPS + digested control of fruits), were as follows: 45.63% by BBs, 40.08% by BKs, and 31.24% by BNs. Figure 2C shows the negative (RPMI‐treated) and positive (LPS‐treated) controls for LPS‐induced NF‐κB activation in macrophages, highlighting significantly higher NF‐κB activation in LPS‐activated macrophages compared to the untreated control. Moreover, NF‐κB activation in untreated macrophages was not significantly different from macrophages treated with basolateral conditioned media from Caco‐2 cells that had been coincubated with digested fruits or control and palm oil, indicating that the digested fruits/control likely contained minimal levels of LPS. Indeed, the final LPS concentrations in the medium given to Caco‐2 cells were comparable across all LPS‐containing treatment conditions (∼0.51 mg/mL), as shown in Supporting Information Table S2. The LPS quantified in the digested fruits or control contributed minimally, representing only 2.28% on average of the total LPS in the final model. Based on the cell viability WST‐1 assay, basolateral conditioned media from Caco‐2 cells did not induce cytotoxicity in macrophages when compared to untreated macrophages (Figure 2D). Although not statistically significant, LPS‐treated cells showed a decreasing trend in cell viability compared to untreated macrophages. Altogether, these results show that digested fruits reduce palm oil + LPS‐mediated NF‐κB activation in macrophages in the intestinal ME cell model.

### 3.3 Digested Blueberries, Blackberries, and Bananas Reduce Palm Oil‐Induced Chylomicron Secretion in Caco‐2 Cells

**FIGURE 3:**
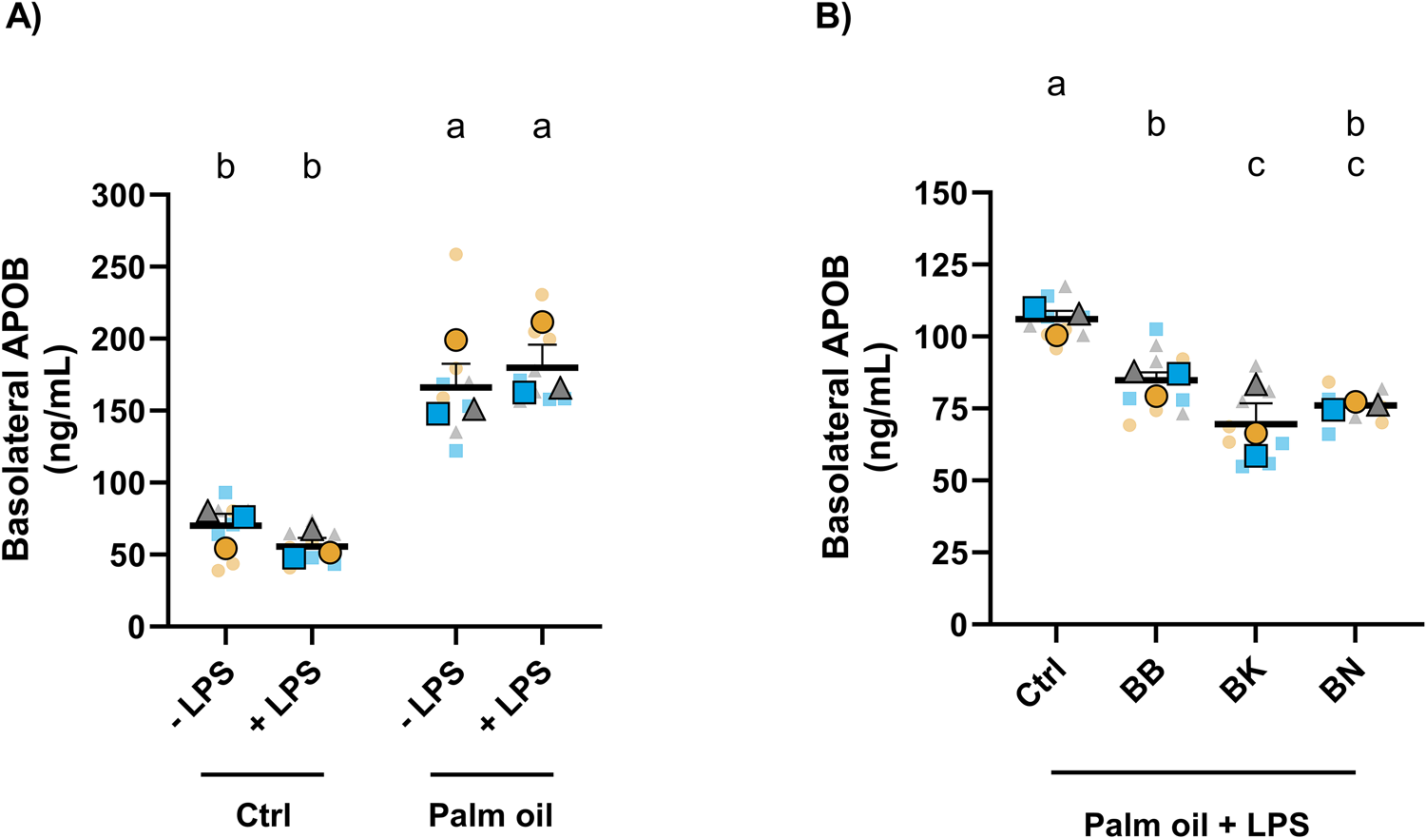
Basolateral APOB levels in Caco‐2 cells (A) upon exposure of digested control or palm oil, without or with LPS; (B) upon exposure of digested palm oil with LPS and digested fruits (BB, BK, or BN) or control. Note that the absolute values of (A, B) are not directly comparable. Statistical differences were determined using a random block two‐way (A) or a one‐way (B) ANOVA to account for differences due to Caco‐2 cell passage number. Post hoc pairwise comparisons were performed using Fisher's LSD test. Different letters (a–c) indicate a significant difference between experimental groups (*p* ≤ 0.05), as determined by ANOVA. Data are presented as the mean ± SEM from three technical and three biological replicates. ANOVA, analysis of variance; APOB, apolipoprotein B; BB, blueberry; BK, blackberry; BN, banana; Fisher's LSD, Fisher's Least Significant Difference; SEM, standard error of the mean.

The molecular mechanisms underlying the suppressive effects of digested fruits on palm oil + LPS‐mediated NF‐κB activation in macrophages were next examined. The analysis focused on transcellular mechanisms, specifically lipid‐induced LPS translocation across enterocytes, which may be mediated by a chylomicron‐dependent pathway [7, 11, 12]. To confirm chylomicron formation in the Caco‐2 cell model, the chylomicron marker APOB [43] was measured in the basolateral conditioned media from Caco‐2 cells that had been coincubated with digested control or palm oil, without or with LPS, for 24 h (same samples as Figure 1). Results showed a significant increase in basolateral APOB levels in Caco‐2 cells that had been treated with digested palm oil compared to control, regardless of LPS (Figure 3A). On average, LPS‐free exposures increased basolateral APOB levels by 2.36‐fold relative to the digested control, and LPS‐containing exposures resulted in a 3.24‐fold increase. After confirming palm oil‐induced chylomicron secretion in the Caco‐2 cell model, the effects of digested fruits on reducing chylomicron levels in Caco‐2 cells cotreated with palm oil and LPS (mimicking a ME context) were assessed. Notably, addition of BBs, BKs, and BNs to digested palm oil with LPS significantly reduced basolateral APOB levels compared to the digested control of fruits, with average decreases of 20.12%, 34.49%, and 28.33%, respectively (Figure 3B) (same samples as Figure 2A,B). Collectively, these findings show the potential of digested fruits in reducing palm oil‐induced chylomicron secretion in Caco‐2 cells under ME conditions.

### 3.4 Chylomicrons Play a Minimal Role in LPS Translocation Across a Confluent Caco‐2 Cell Monolayer

**FIGURE 4:**
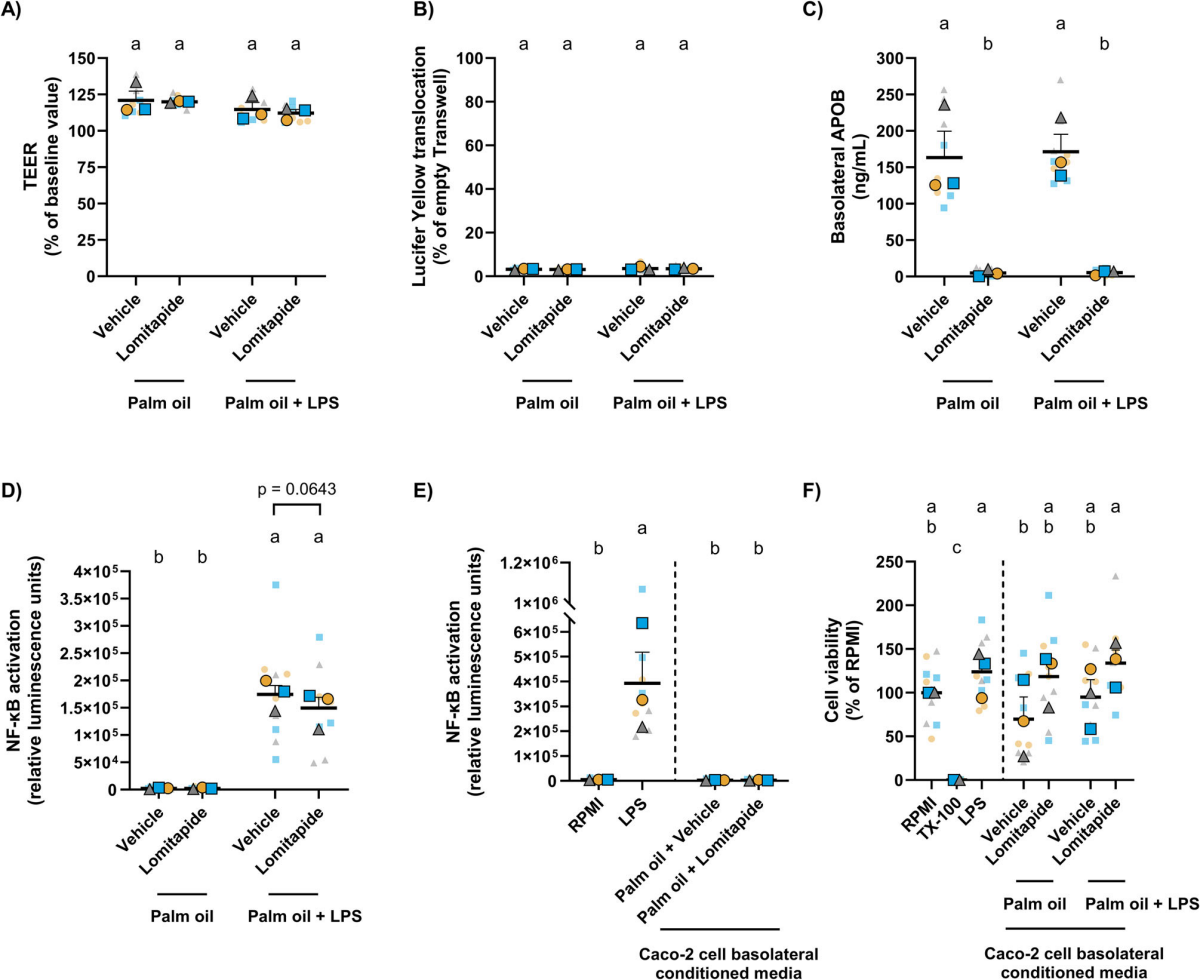
Effects of Lomitapide or vehicle and digested palm oil without or with LPS on Caco‐2 cells. Intestinal permeability was assessed by (A) TEER and (B) LY assay. (C) Basolateral APOB levels in Caco‐2 cells. (D) NF‐κB activation in THP1‐Lucia NF‐κB macrophages after 24 h exposure to basolateral conditioned media from Caco‐2 cells that had been cotreated with Lomitapide or vehicle and digested palm oil without or with LPS for 24 h. (E) NF‐κB activation in macrophages after 24 h exposure to RPMI, LPS (30 ng/mL), or basolateral conditioned media from Caco‐2 cells that had been coincubated with digested palm oil and Lomitapide or vehicle. (F) Viability of macrophages treated with RPMI, TX‐100 (1%), LPS (30 ng/mL), or basolateral conditioned media from Caco‐2 cells that had been cotreated with Lomitapide or vehicle and digested palm oil without or with LPS. Statistical differences were determined using a random block two‐way (A, C, D) or one‐way (E) ANOVA to account for differences due to Caco‐2 cell passage number; or an ordinary two‐way (B) or one‐way (F) ANOVA. Post hoc pairwise comparisons were performed using Fisher's LSD test. Different letters (a–c) indicate a significant difference between experimental groups (*p* ≤ 0.05), as determined by ANOVA. Data are presented as the mean ± SEM from three technical and three biological replicates. ANOVA, analysis of variance; APOB, apolipoprotein B; Fisher's LSD, Fisher's Least Significant Difference; LY, Lucifer Yellow; NF‐κB, nuclear factor kappa B; RPMI, Roswell Park Memorial Institute; SEM, standard error of the mean; TEER, transepithelial electrical resistance; TX‐100, Triton‐X 100.

To examine whether LPS was translocated via chylomicrons in the model, Caco‐2 cells were coincubated with digested palm oil, without or with LPS, and the chylomicron synthesis inhibitor Lomitapide (targets microsomal triglyceride transfer protein [MTTP]) [29, 44, 45, 46] or vehicle (0.1% DMSO) for 24 h. After incubation, intestinal permeability was assessed by TEER (Figure 4A) and LY (Figure 4B). On average, TEER values were maintained at 116.9 ± 4.2%, and basolateral LY at 3.3 ± 0.3%, indicating high confluency of the Caco‐2 cell monolayer. Basolateral APOB levels were significantly reduced after coincubation of palm oil with Lomitapide compared to vehicle, regardless of LPS addition (Figure 4C). On average, Lomitapide reduced basolateral APOB values below 3.0%. Subsequently, basolateral conditioned media from Caco‐2 cells were exposed to THP1‐Lucia NF‐κB macrophages, and NF‐κB activation in macrophages was used as a proxy measurement of LPS translocation across the Caco‐2 cell monolayer (Figure 4D). Conditioned medium from Caco‐2 cells that had been cotreated with digested palm oil, LPS, and vehicle resulted in a significantly increased activation of the NF‐κB pathway in macrophages compared to conditioned medium from Caco‐2 cells that had been cotreated with digested palm oil and vehicle (Figure 4D). Interestingly, Lomitapide modestly reduced macrophage NF‐κB activation (by an average of 14.36%) in Caco‐2 cells cotreated with palm oil and LPS, tending toward statistical significance (*p* = 0.0643). Figure 4E shows the negative (RPMI‐treated) and positive (LPS‐treated) controls for LPS‐induced NF‐κB activation in macrophages, demonstrating significantly elevated NF‐κB activation in LPS‐treated macrophages compared to untreated controls. Viability of macrophages across the various treatments did not significantly differ from untreated cells (representing 100% viability) (Figure 4F). Altogether, these results suggest a minimal role of chylomicrons in palm oil‐induced translocation of LPS across Caco‐2 cells and subsequent NF‐κB activation in macrophages.

### 3.5 Digested Berries Downregulate the Expression of Genes Involved in Chylomicron‐ and Transcytosis‐Dependent Mechanisms in Caco‐2 Cells

**FIGURE 5:**
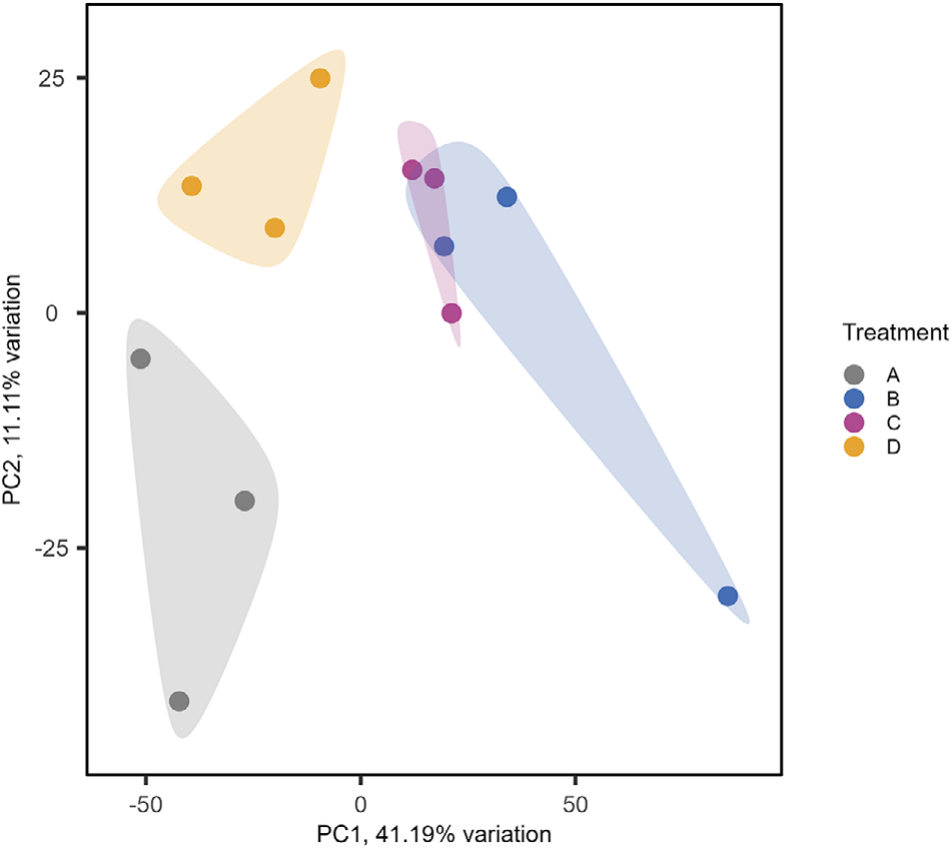
Principal component analysis (PCA) plot of Caco‐2 cell gene expression. Caco‐2 cells were exposed to digested palm oil and LPS with (A) digested control of the fruits, (B) digested blueberries, (C) digested blackberries, or (D) digested bananas, for 24 h. Shaded areas represent convex hulls enclosing the data points of each treatment group. Data points correspond to biological replicates, each derived from a pool of three technical replicates.

To gain insight into alternative molecular mechanisms underlying the anti‐inflammatory effects of digested fruits on palm oil + LPS‐induced NF‐κB signaling in the coculture cell model, RNA sequencing on Caco‐2 cells was performed. The analysis focused on transcriptome data from Caco‐2 cells cotreated with digested palm oil, LPS, and digested fruits or digested control of the fruits for 24 h. The principal component analysis (PCA) plot illustrates the gene expression patterns across the different exposures (Figure 5). Clear overlapping clusters were observed along principal component 1 (accounting for 41.19% of the variation), with the digested control and BN samples forming one cluster, and the BB and BK samples grouping together in a separate cluster.

**FIGURE 6:**
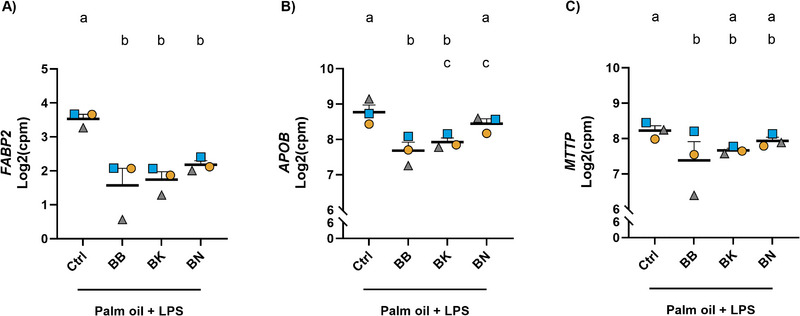
Caco‐2 cell gene expression profiles of (A) *FABP2*, (B) *APOB*, and (C) *MTTP* upon treatment with digested palm oil, LPS, and digested control, BB, BK, or BN. Data are expressed as Log2(cpm), counts per million reads. Different letters (a–c) indicate a significant difference between experimental groups (FDR ≤ 0.05). Data points correspond to biological replicates, each derived from a pool of three technical replicates, ± SEM. *APOB*, apolipoprotein B; BB, blueberry; BK, blackberry; BN, banana; *FABP2*, fatty acid binding protein 2; FDR, false discovery rate; *MTTP*, microsomal triglyceride transfer protein; SEM, standard error of the mean.

Subsequent analysis examined whether molecular pathways involved in lipid‐mediated LPS translocation in the intestine, specifically chylomicron‐ and transcytosis‐mediated mechanisms, were suppressed following exposure to the digested fruits. Thus, changes in the expression of genes involved in the Kyoto Encyclopedia of Genes and Genomes (KEGG) fat digestion and absorption pathway (KEGG, hsa04975), which includes genes implicated in chylomicron metabolism, were analyzed. Genes showing a significant effect (FDR ≤ 0.05) in at least one fruit treatment versus the control were selected for analysis. Interestingly, BB treatment significantly modulated the expression of many genes in this pathway. BK induced a moderate effect, while BN resulted in a few significantly differentially expressed genes, compared to the digested control (Supporting Information Table S3). Notably, BB significantly downregulated crucial genes for chylomicron assembly [43, 47], including fatty acid binding protein 2 (*FABP2*, intestinal fatty acid binding protein), *APOB*, and *MTTP* (Figure 6). In Supporting Information Figure S1, the PCA biplot for chylomicron‐associated genes (as listed in Supporting Information Table S3) is shown. Taken together, these results point to a significant role of palm oil in LPS translocation and suggest that fruits may mitigate this process by modulating lipid uptake, intracellular metabolism, and excretion.

Transcytosis, a proposed mechanism for LPS translocation across the intestinal epithelium [8, 14, 48], involves coordinated cellular processes including endocytosis, endosomal trafficking, Golgi transport, and exocytosis via extracellular vesicles (EVs) [13, 49, 50, 51]. Because no KEGG pathway comprehensively lists all genes involved in these processes, key rate‐limiting transcytosis genes were identified based on current literature. Interestingly, several of these genes showed a significant effect (FDR ≤ 0.05) and a Log2 fold change ≥ 1 in at least one fruit treatment relative to the control (Table 1). Supporting Information Table S4 includes the identified genes with a Log2 fold change relative to control ≤ 1. Given that multiple mechanisms for endocytosis have been described [49], only genes associated with clathrin‐mediated transcytosis (the primary [52, 53] and most well‐understood [49, 50] transcytotic pathway) were selected for analysis. Results showed that multiple clathrin‐related transcytotic genes were significantly modulated by digested BBs, and to a lesser extent by BKs, while BNs exhibited no significant effects. Interestingly, BBs significantly reduced the expression profiles of key genes involved in endocytosis [50] such as intersectin 1 (*ITSN1*)/intersectin 2 (*ITSN2*) [49, 50, 54], stonin 1 (*STON1*) [49], FCH and mu domain containing endocytic adaptor 2 (*FCHO2*) [50], myosin VI (*MYO6*) [49], clathrin heavy chain linker domain containing 1 (*CLHC1*) [55], and dynamin 1‐like (*DNM1L*) [56, 57]. Similarly, BB negatively regulated genes involved in endosomal trafficking [58] such as early endosome antigen 1 (*EEA1*) [53], RAB9B, member RAS oncogene family (*RAB9B*) [58], WASH complex subunit 4 (*WASHC4*) [56, 59], and adaptor related protein complex 3 subunit beta 1 (*AP3B1*) [50, 58]; and Golgi transport [58] including the golgins golgin A8 family member A (*GOLGA8A*)*/*golgin A8 family member B (*GOLGA8B*) [60] and *GOLGA4* [61, 62]. Exocytosis was also negatively regulated by the BB exposure. Particularly, the genes *HSP90 AA1/B1* [63], RAB27B, member RAS oncogene family (*RAB27B*) [53], and charged multivesicular body protein 2B (*CHMP2B*) [64]. In Supporting Information Figure S2, the PCA biplot for transcytosis‐associated genes (as described in Table 1 and Supporting Information Table S4) is shown. Interestingly, Supporting Information Figure S3 shows volcano plots comparing each fruit to the control, highlighting that the transcytotic genes *EEA1*, *GOLGA4*, and *ITSN2* were among the most significantly downregulated in the BB and BK exposures.

**TABLE 1:** Caco‐2 cell gene expression profiles of transcytosis‐associated genes following treatment with digested palm oil, LPS, and digested BB, BK, or BN compared to Ctrl.

Gene name	BB vs. Ctrl LPS		BK vs. Ctrl LPS		BN vs. Ctrl LPS		Cellular compartment/transcytotic pathway
	Log2FC	FDR	Log2FC	FDR	Log2FC	FDR	
*GOLGA8B*	−2.671	**	−0.912	ns	−0.775	ns	Golgi
*EEA1*	−2.287	****	−2.260	****	−0.659	ns	Endosomes
*HSP90AA1*	−2.157	***	−1.623	**	−0.214	ns	Exosomes
*RAB9B*	−2.101	**	−1.244	*	−0.834	ns	Endosomes/golgi
*GOLGA8A*	−2.055	**	−1.341	*	−0.934	ns	Golgi
*RAB27B*	−1.996	**	−0.983	ns	−0.586	ns	Exosomes
*GOLGA4*	−1.924	****	−1.919	****	−0.459	ns	Golgi
*ITSN2*	−1.833	***	−1.775	***	−0.160	ns	Endocytosis
*HSP90B1*	−1.595	***	−1.186	**	−0.143	ns	Exosomes
*STON1*	−1.575	*	−0.298	ns	−0.280	ns	Endocytosis
*WASHC4*	−1.548	**	−1.278	**	−0.323	ns	Endosomes
*FCHO2*	−1.498	***	−1.307	**	−0.204	ns	Endocytosis
*MYO6*	−1.313	**	−0.987	*	0.038	ns	Endocytosis
*CLHC1*	−1.239	**	−0.802	ns	−0.366	ns	Endocytosis
*DNM1L*	−1.127	**	−0.826	*	−0.365	ns	Endocytosis
*ITSN1*	−1.064	**	−0.854	*	−0.081	ns	Endocytosis
*AP3B1*	−1.035	**	−0.680	*	−0.138	ns	Endosomes
*CHMP2B*	−1.025	**	−0.443	ns	0.045	ns	Exosomes

*Note*: Data are expressed as Log2 fold change (FC) relative to control. Statistical significance was defined as follows: ns (FDR > 0.05), * (FDR ≤ 0.05), ** (FDR ≤ 0.01), *** (FDR ≤ 0.001), **** (FDR ≤ 0.0001).

Abbreviations: AP3B1, adaptor related protein complex 3 subunit beta 1 [58]; BB, blueberry; BK, blackberry; BN, banana; CHMP2B, charged multivesicular body protein 2B [64]; CLHC1, clathrin heavy chain linker domain containing 1 [94]; Ctrl, control; DNM1L, dynamin 1‐like [94]; EEA1, early endosome antigen 1 [53]; FCHO2, FCH and mu domain containing endocytic adaptor 2 [50]; GOLGA4, golgin A4 [61]; GOLGA8A, golgin A8A [60]; GOLGA8B, golgin A8B [60]; HSP90AA1, heat shock protein 90 kDa alpha family class A member 1 [63]; HSP90B1, heat shock protein 90 kDa beta family member 1 [63]; ITSN1, intersectin 1 [49]; ITSN2, intersectin 2 [49]; MYO6, myosin VI [49]; RAB27B, RAB27B, member RAS oncogene family [53]; RAB9B, RAB9B, member RAS oncogene family [58]; STON1, stonin 1 [49]; WASHC4, WASH complex subunit 4 [59].

**FIGURE 7:**
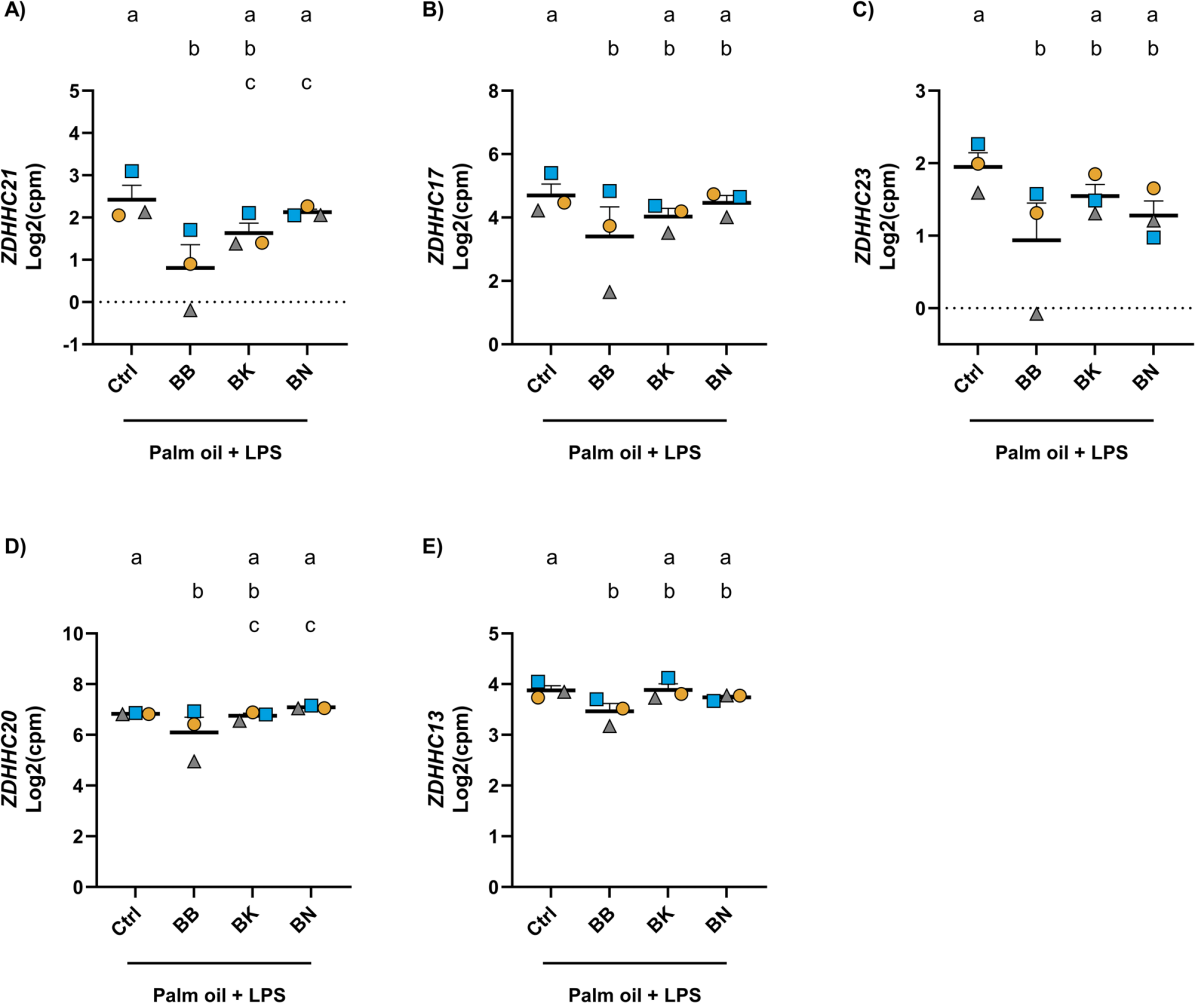
Caco‐2 cell gene expression profiles of (A) *ZDHHC21*, (B) *ZDHHC17*, (C) *ZDHHC23*, (D) *ZDHHC20* and (E) *ZDHHC13* upon treatment with digested palm oil, LPS, and digested control, BB, BK, or BN. Data are expressed as Log2(cpm), counts per million reads. Different letters (a–c) indicate a significant difference between experimental groups (FDR ≤ 0.05). Data points correspond to biological replicates, each derived from a pool of three technical replicates, ± SEM. BB, blueberry; BK, blackberry; BN, banana; FDR, false discovery rate; SEM, standard error of the mean; *ZDHHC*, zinc finger aspartate‐histidine‐histidine‐cysteine.

In the context of transcytosis and EV secretion, palmitoylation has been recently suggested as a novel regulator of EV formation [65]. Palmitoylation is a post‐translational modification in which a palmitate is attached to a target protein by transmembrane, zinc finger aspartate‐histidine‐histidine‐cysteine (zDHHC)‐motif‐containing palmitoyl acyltransferases (reviewed by Yang et al. [66] and Ko and Dixon [67]). Interestingly, BBs significantly downregulated multiple *ZDHHC* genes when compared to the control. In particular, *ZDHHC 21*, *17*, *23*, *20*, and *13*. However, BKs and BNs did not show statistically significant effects (Figure 7). Collectively, these findings suggest the potential of BB exposure in reducing transcytosis in Caco‐2 cells.

## 4 Discussion

This study examined the effects and potential underlying molecular mechanisms of whole digested BBs, BKs, and BNs on intestinal ME using an in vitro coculture cell model of intestinal Caco‐2 cells and THP1‐Lucia NF‐κB macrophages. This coculture cell model was optimized based on the Caco‐2 cell Transwell system for ME described by Tomassen et al. [11]. To this end, activation of the THP1‐Lucia NF‐κB macrophage cell line was incorporated as a read‐out for LPS translocation across the Caco‐2 cell monolayer. The apical LPS concentration Caco‐2 cells were exposed to was increased to 0.5 mg/mL to achieve consistent basolateral macrophage activation.

Our Caco‐2 cell model showed palm oil‐induced LPS translocation to the basolateral compartment, as indicated by significantly higher basolateral LPS levels following coincubation with palm oil and LPS, compared to the digested control with LPS (∼572‐fold increase). Palm oil‐induced LPS transport may be mediated via a transcellular pathway, as shown by the limited paracellular permeability, with only ∼2.6% of the 0.4 kDa LY translocating the Caco‐2 cell monolayer. This suggests that the significantly larger LPS molecule (10–20 kDa [68]) was unlikely to translocate via a paracellular route. These findings are in agreement with previous studies showing transcellular lipid‐induced LPS transport in Caco‐2 cells [7, 11]. However, small amounts of LPS were also detected in the basolateral compartment of Caco‐2 cells treated with digested control and LPS, suggesting LPS may be transported through other unknown mechanisms.

To investigate the biological effects of palm oil‐induced translocated LPS on immune cell activation, the basolateral conditioned media from Caco‐2 cells were added to THP1‐Lucia NF‐κB macrophages, followed by assessment of the NF‐κB signal transduction pathway. LPS specifically binds to Toll‐like receptor 4 (TLR4) and mediates signal transduction of NF‐κB, a key regulator of inflammation [18, 69]. Importantly, palm oil‐induced translocated LPS triggered NF‐κB activation in macrophages, confirming its bioactivity. These results suggest functional immune cell activation and align with the findings of Tomassen et al. [11], who showed that lipid‐induced translocated LPS binds to TLR4 in HEK‐Blue hTLR4 cells. Functional measurements for LPS are crucial for research in ME [70], as none of the available detection assays (i.e., Limulus amebocyte lysate [71] and ELISA [72]) conclusively demonstrate whether the detected LPS molecules can stimulate host cells.

Based on literature [73, 74, 75], it was hypothesized that polyphenolics might mediate the effects of berries, given their content of ∼11 mg of gallic acid equivalents (GAEs)/g dry weight [73]. Hence, BN pulp was selected as a (negative) control fruit due to its low polyphenolic content (∼2 mg GAE/g dry weight) [74, 75] and its botanical classification as a berry. It was observed that the basolateral conditioned media from Caco‐2 cells that had been exposed to the digested fruits reduced macrophage NF‐κB activation, with BB showing the most significant and consistent effects. These findings suggest that BBs, BKs, and BNs may alleviate palm oil‐induced LPS translocation in Caco‐2 cells. The observed reduction in macrophage NF‐κB activation by BNs suggests that bioactives other than polyphenolics may contribute to mitigating ME. Regarding the effects of berries, the results align with findings by Anhê et al., who reported reduced plasma LPS levels in mice fed with an HFHS diet supplemented with berry extract [19]. Similarly, Gasparrini et al. reviewed the direct anti‐inflammatory effects of berries on in vitro LPS‐stimulated inflammatory models [76]. However, there is limited literature regarding the detailed mechanism(s) of digested fruits on LPS translocation across the intestinal epithelium in the context of ME. This study is the first to report the effects of digested whole berries and BNs on lipid‐induced LPS translocation across a Caco‐2 cell monolayer.

From existing studies [1, 7, 11, 12], it was initially hypothesized that chylomicrons mediate LPS translocation across enterocytes. The Caco‐2 cell model showed secretion of palm oil‐induced chylomicrons, as palm oil‐treated Caco‐2 cells released significantly higher levels of basolateral APOB compared to the control. These findings are consistent with those of Luchoomun and Hussain [77] and Ghoshal et al. [7], who showed lipid‐induced chylomicron secretion in Caco‐2 cells. Interestingly, the digested fruits significantly reduced basolateral APOB secretion in Caco‐2 cells compared to the control, suggesting their potential to reduce chylomicron release in the intestine. Consistently, RNA sequencing analysis showed similar results since key genes for chylomicron assembly [43, 47] such as *FABP2*, *APOB*, and *MTTP* were significantly reduced after incubation with the digested fruits; BB elicited the most prominent effects, followed by BKs and then BNs. Similarly, other studies have shown the reductive effects of berry‐derived micronutrients [78, 79, 80, 81], such as anthocyanins [82], resveratrol [83], and niacin [84] on APOB‐48. Thus, consuming berries or BNs may prevent conditions linked to elevated postprandial chylomicron levels, including insulin resistance [85], obesity [86], and atherosclerosis [43].

Furthermore, almost complete blockage of chylomicrons by Lomitapide (∼97%) led to only an ∼14% decrease in NF‐κB macrophage activation, almost reaching statistical significance (*p* = 0.0643). Similarly, Tomassen et al. found a suppressive effect on LPS translocation across Caco‐2 cells following chylomicron inhibition with Pluronic L‐81, although it did not reach statistical significance [11]. Our findings align with Akiba et al., who showed that LPS transport follows a biphasic pattern in a rodent model, with the majority of translocated LPS delivered to the liver via the portal vein, while a smaller fraction is transported through the canonical chylomicron pathway via the lymphatic system [48]. Briefly, Akiba et al. showed that in vivo intraduodenal perfusion of oleic acid and taurocholate rapidly increased the presence of coperfused fluorescein isothiocyanate (FITC)‐LPS into the portal vein (∼60%), followed by a gradual increase of FITC‐LPS into the lymph (∼1%) [48]. Hence, chylomicrons contribute only minimally, suggesting that another transcellular, secretory transport mechanism is responsible for LPS translocation and the observed effects of the berries in the model.

Indeed, LPS has been proposed to cross the intestinal epithelium via transcytosis [8, 13, 14, 48]. To examine the effects of digested fruits or control on transcytotic mechanisms, transcriptomic analysis was conducted on Caco‐2 cells that had been cotreated with digested palm oil, LPS, and digested fruit or control. BBs had the most significant impact, substantially downregulating key genes involved in various stages of transcytosis, including *EEA1* [53], *ITSN1/2* [49, 50, 54], *CLHC1* [55], and *GOLGA8* [60]. In contrast, BKs induced modest effects, while BNs showed minimal to no significant influence. Altogether, clathrin‐mediated transcytosis may represent an alternative mechanism through which BBs, and to some extent BKs, may mitigate ME. However, the involvement of additional transcytosis pathways beyond clathrin‐mediated mechanisms cannot be excluded [87].

**FIGURE 8:**
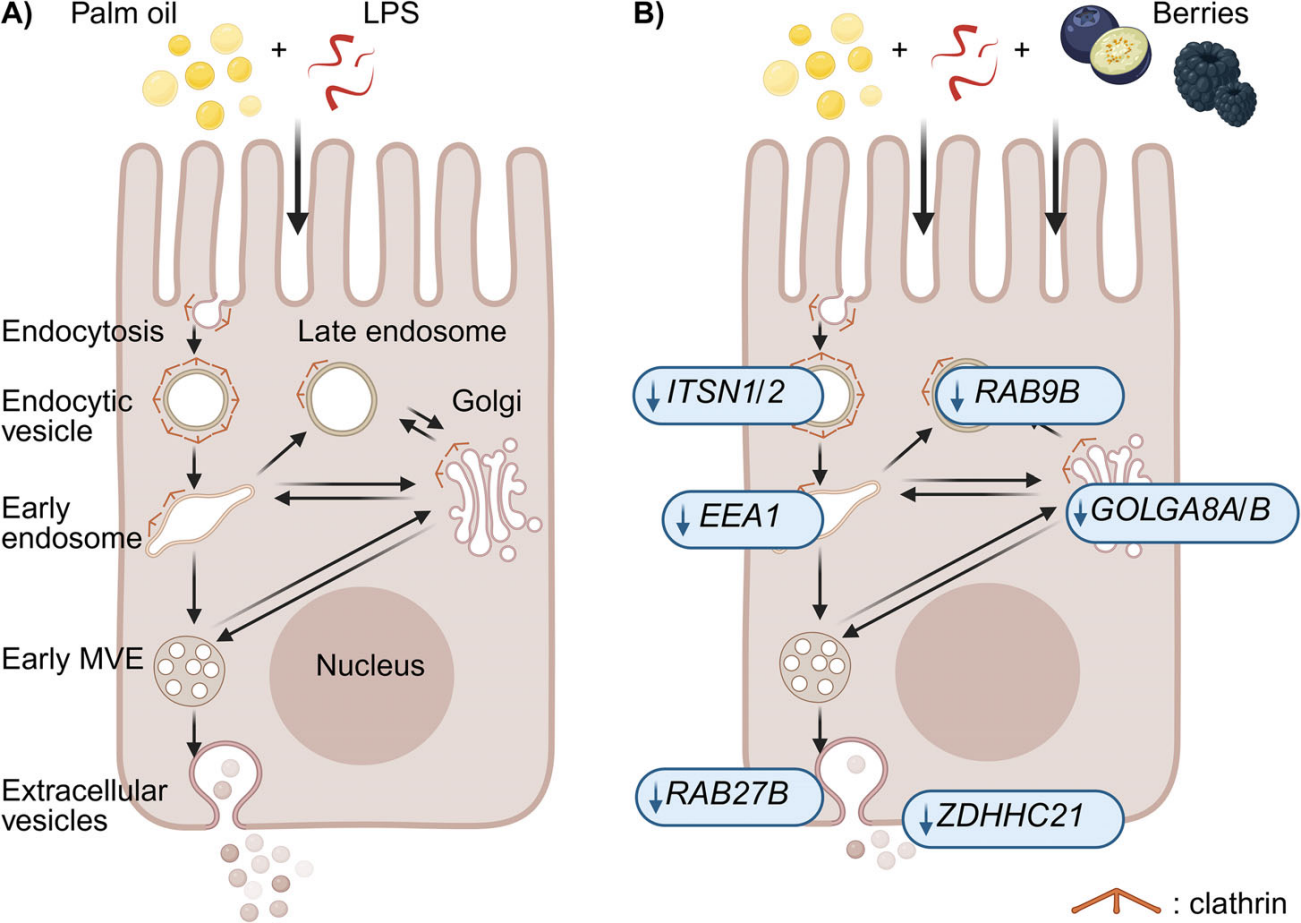
Schematic representation of (A) clathrin‐mediated transcytosis in an enterocyte, adapted from Raiborg et al. [93] and Johannes et al. [58]. Clathrin is shown in orange. (B) Key transcytotic genes downregulated by blue‐ and blackberries during coexposure with digested palm oil and LPS in Caco‐2 cells. Image generated in BioRender. MVE, multivesicular endosome.

Another potential regulator of transcytosis and EV formation is palmitoylation [66, 67]. Multiple membrane proteins involved in EV biogenesis undergo palmitoylation [65, 88, 89], a modification that increases protein lipophilicity and membrane localization, further influencing exocytosis [66, 90]. Interestingly, only BBs significantly downregulated several *ZDHHC* genes, including *Z* *DHHC21*, *17*, *23*, *20*, and *13*, compared to the control. Notably, *ZDHHC21* exhibited the most statistically significant and strongest reducing effect (Log2FC = −1.5, FDR ≤ 0.01), compared to the control. Remarkably, Yang et al. [65] reported a key role of ZDHHC21 in EV formation. They showed  significantly reduced plasma EVs in mice with functional deficiency of ZDHHC21 compared to wild‐type mice [65]. Collectively, lipid‐induced LPS translocation across the intestinal epithelium may involve transcytotic mechanisms, which could be partly regulated by palmitoylation. BBs may reduce LPS translocation by downregulating key *ZDHHC* genes, such as *ZDHHC21*. A schematic representation of clathrin‐mediated transcytosis and key findings are summarized in Figure 8.

## 5 Limitations

In vitro models may oversimplify the biological processes occurring in the in vivo phenomenon of ME, so findings should be interpreted with some caution and ideally validated in vivo. This study examined the effects of digested whole berries and BNs, offering greater physiological relevance, but did not analyze individual polyphenolics or other constituents [91]. The lack of a chemical analysis of the digested fruits might be considered a limitation. Yet, although of interest, it should be emphasized that a detailed analysis of the composition of the fruits can merely provide leads and mostly speculation. Berries contain thousands of different compounds, making it extremely difficult to pinpoint any specific bioactives that might be responsible for the observed effects. Future studies using fractionation or purified compounds will be necessary to identify the relevant bioactives [91]. Only pancreatic, and no gastric, lipase was added in the gastrointestinal digestion model [11] because pancreatic lipase is the predominant enzyme in gastrointestinal lipolysis of dietary triacylglycerols, accounting for three‐quarters of fat digestion in healthy humans [92]. Finally, this study did not include a chemical analysis of the digested palm oil, which would allow further characterization of digestion products.

## 6 Concluding Remarks

A novel and physiologically relevant in vitro human intestinal‐immune cell model was developed to study the effects of digested whole foods or dietary compounds in the presence of LPS. This model was used to examine the effects of digested whole BBs, BKs, and BNs on intestinal barrier function and immune response in macrophages. The digested fruits alleviated palm oil‐induced LPS translocation across a Caco‐2 cell monolayer, likely via clathrin‐mediated transcytosis and a minimal chylomicron‐dependent mechanism. Notably, BBs elicited the most significant and consistent effects, followed by BKs, with BNs showing the least impact. These findings suggest that the anti‐inflammatory support of fruit‐rich diets may be mediated by their preventive effect on ME.

## Funding

The study is part of MOCIA (Maintaining Optimal Cognitive function In Ageing), an NWO Crossover grant (MOCIA 17611) from the Dutch Research Council (Nederlandse Organisatie voor Wetenschappelijk Onderzoek [NWO]) (see https://mocia.nl/scientific/).

## Conflicts of Interest

The authors declare no conflicts of interest.

## Supporting information




**Supporting Information**: mnfr7031‐sup‐0001‐SuppMat.docx.

## Data Availability

All data generated or analyzed during this study are included in this article.
