# Supplementary material for: Blueberries Reduce Palm Oil‐Induced Metabolic Endotoxemia in an In Vitro Human Intestinal‐Immune Cell Model
Source: Mol Nutr Food Res. 2025 Nov 11;69(24):e70311. doi: 10.1002/mnfr.70311 (PMC12700054; doi:10.1002/mnfr.70311)
Supplement: Supplementary file 1 — Supporting Information: mnfr7031‐sup‐0001‐SuppMat.docx. [file MNFR-69-e70311-s001.docx]

**
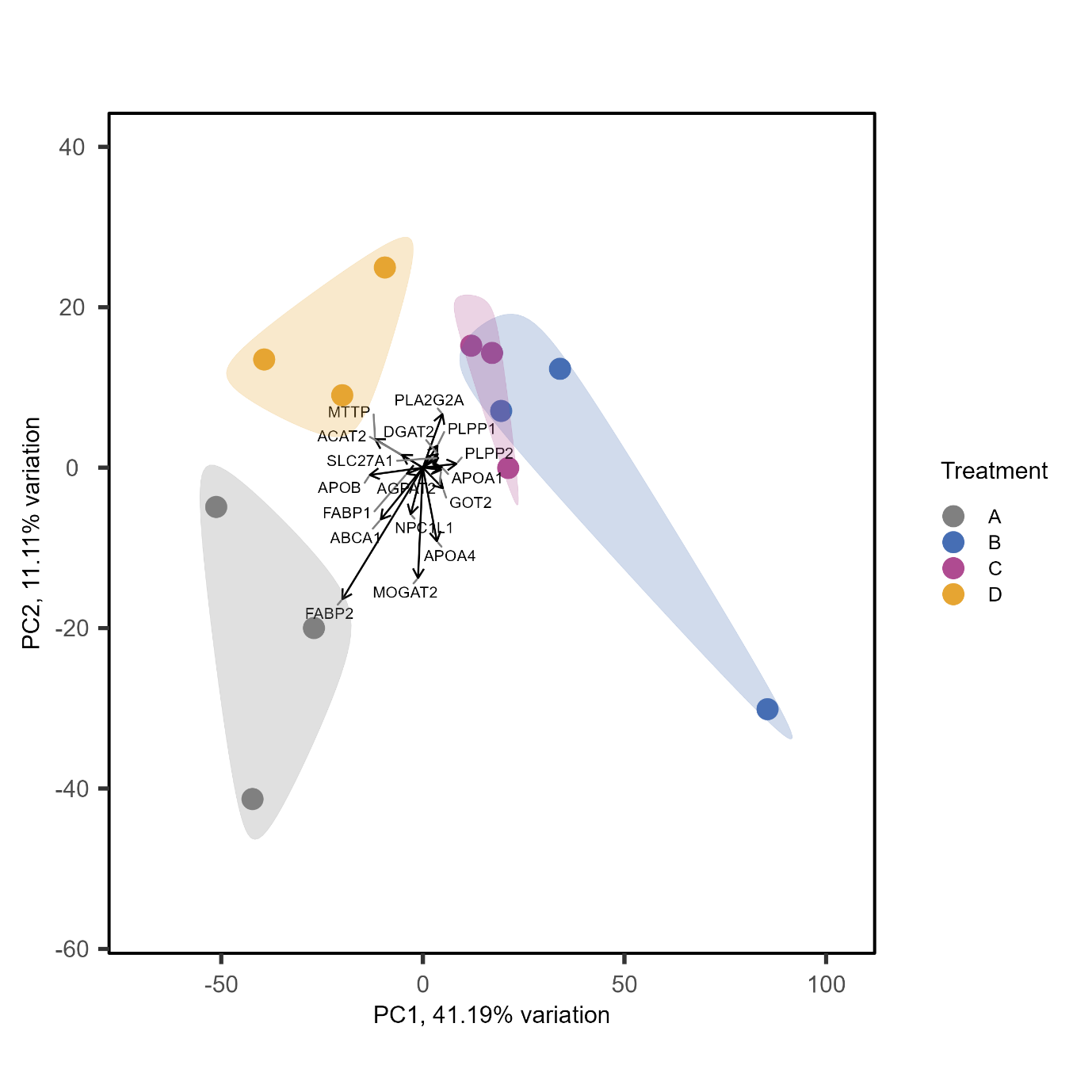
**

**Supplementary Figure 1.** Principal component analysis (PCA) biplot of Caco-2 cell gene expression for chylomicron-associated genes (as described in **Supplementary Table 3**). Caco-2 cells were exposed to digested palm oil and LPS with A) digested control of the fruits, B) digested blueberries, C) digested blackberries, or D) digested bananas, for 24 h. Shaded areas represent convex hulls enclosing the data points of each treatment group. Data points correspond to biological replicates, each derived from a pool of three technical replicates.


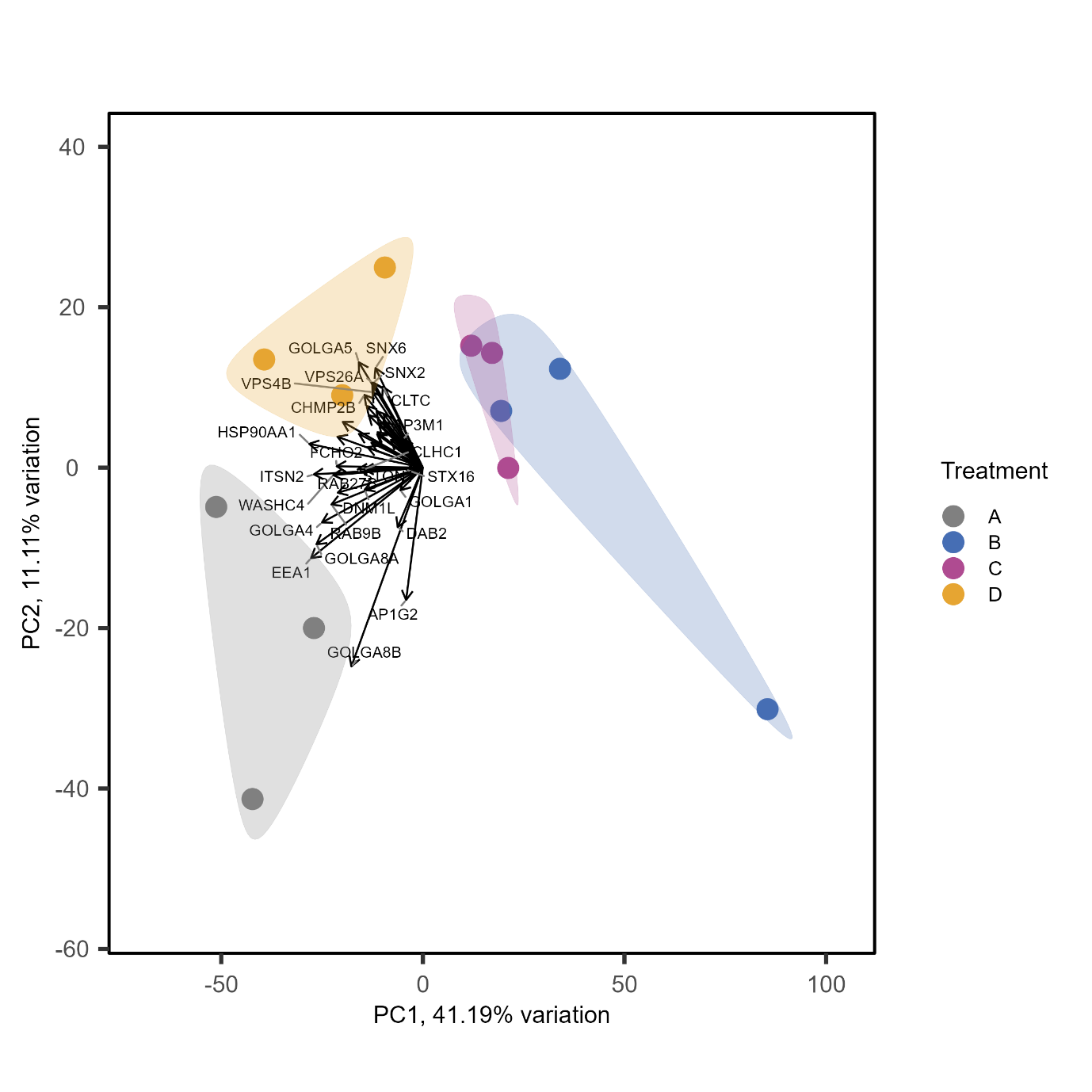


**Supplementary Figure 2.** Principal component analysis (PCA) biplot of Caco-2 cell gene expression for transcytosis-associated genes (as described in **Table 1** and **Supplementary Table 4**). Caco-2 cells were exposed to digested palm oil and LPS with A) digested control of the fruits, B) digested blueberries, C) digested blackberries, or D) digested bananas, for 24 h. Shaded areas represent convex hulls enclosing the data points of each treatment group. Data points correspond to biological replicates, each derived from a pool of three technical replicates.

**
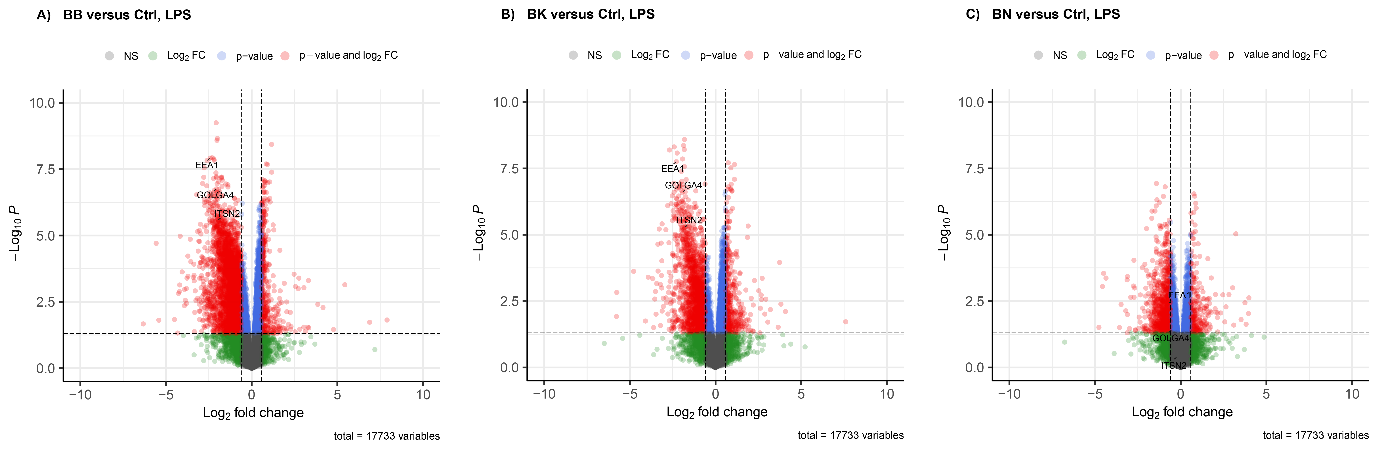
**

**Supplementary Figure 3.** Volcano plots showing differential gene expression in Caco-2 cells treated with digested palm oil, LPS, and digested control (Ctrl), BB, BK, or BN. A) Ctrl vs BB, B) Ctrl vs BK, C) Ctrl vs BN. Data are presented as Log2 fold change versus -Log10 moderated *p* value.

**Supplementary Table 1**: Fatty acid composition profile of the undigested palm oil. Data are presented as relative (%) values.

|  | **Compound** | **Palm oil** |
| --- | --- | --- |
|  |  | **Relative**  **(%)** |
|  | C4:0 Butyric acid | <0.05 |
|  | C6:0 Caproic acid | <0.05 |
|  | C8:0 Caprylic acid | <0.05 |
|  | C10:0 Capric acid | <0.05 |
|  | C10:1 Caproleic acid | <0.05 |
|  | C11:0 Undecanoic acid | <0.05 |
|  | C12:0 Lauric acid | 0.20 |
|  | C12:1 (n-9c) Lauroleic acid | <0.05 |
|  | C13:0 Tridecoic acid | <0.05 |
|  | C14:0 Myristic acid | 0.97 |
|  | C14:1(n-5c) Myristoleic acid | <0.05 |
|  | C15:0 Pentadecanoic acid | 0.05 |
|  | C16:0 Palmitic acid | 44.91 |
|  | C16:1(n-7c) Palmitoleic acid | 0.15 |
|  | C16:1(n-7t) Palmitelaidic acid | <0.05 |
|  | C16:1(n-9c) Hypogeic acid | <0.05 |
|  | C16:1(n-9t) (7E)-7-Hexadecenoic acid | <0.05 |
|  | C16:2(n-4c) Palmitolinoleic acid | <0.05 |
|  | C17:0 Margarinic acid | 0.11 |
|  | C18:0 Stearic acid | 4.44 |
|  | C18:1 (n-12c), cis-12-Octadecenoic acid | <0.05 |
|  | C18:1(n-12t) Petroselaidic acid | <0.05 |
|  | C18:1(n-7c) Vaccenic acid | 0.68 |
|  | C18:1(n-7t) trans-Vaccenic acid | <0.05 |
|  | C18:1(n-9c) Oleic acid | 38.81 |
|  | C18:1(n-9t) Elaidinic acid | <0.05 |
|  | C18:2 (9c,11t) 9(Z),11(E)-Octadecadienoic acid | <0.05 |
|  | C18:2(6c,9t) 9(E),12(Z)-Octadecadienoic acid | <0.05 |
|  | C18:2(n-6c) Linolic acid | 7.92 |
|  | C18:3(n-3c) alpha-Linolenic acid | 0.12 |
|  | C18:3(n-6c) gamma-Linolenic acid | <0.05 |
|  | C18:4(n-3c) Steridonic acid | <0.05 |
|  | C19:0 Nonadecylic acid | <0.05 |
|  | C19:1(n-12t) (7E)-7-Nonadecenoic acid | 0.16 |
|  | C20:0 Arachidic acid | 0.37 |
|  | C20:1(n-15c) (5Z)-5-Eicosenoic acid | <0.05 |
|  | C20:1(n-9c) Gondoic acid | 0.14 |
|  | C20:2(n-6c) (11Z,14Z)-Eicosadienoic acid | <0.05 |
|  | C20:3(n-3c) (17Z)-17-Eicosatrienoic acid | <0.05 |
|  | C20:3(n-6c) (14Z)-14-Eicosatrienoic acid | <0.05 |
|  | C20:4(n-3c) cis-8,11,14,17-Eicosatetraenoic acid | <0.05 |
|  | C20:4(n-6c) Arachidonic acid | <0.05 |
|  | C20:5(n-3c) Eicosapentaenoic acid | <0.05 |
|  | C21:0 Heneicosanoic acid | <0.05 |
|  | C22:0 Behenic acid | 0.06 |
|  | C22:1(n-9c) Erucic acid | <0.05 |
|  | C22:2(n-6c) Docosadienoic acid | <0.05 |
|  | C22:4(n-6c) Adrenic acid | <0.05 |
|  | C22:5(n-3c) cis-19-Docosapentaenoic acid | <0.05 |
|  | C22:5(n-6c) Osbond acid | <0.05 |
|  | C22:6(n-3c) Docosahexaenoic acid | <0.05 |
|  | C23:0 Tricosanoic acid | <0.05 |
|  | C24:0 Lignoceric acid | 0.07 |
|  | C24:1(n-9c) Nervonic acid | <0.05 |
| Summary | Saturated fatty acids | 51.18 |
|  | Mono-unsaturated fatty acid | 39.94 |
|  | Poly-unsaturated fatty acids | 8.04 |
|  | Total | 99.16 |

**Supplementary Table 2**: Total LPS concentrations (mg/mL) in the apical compartment of Caco-2 cells under the indicated treatments. Data are presented as the mean ± SD from three technical replicates.

| **Digested treatments** | | **Final LPS concentration (mg/mL)** | | | |
| --- | --- | --- | --- | --- | --- |
|  |  | **Without LPS** | | **With LPS** | |
|  |  | **Mean** | **SD** | **Mean** | **SD** |
| **Oils** | Control | 0.000 | 2.0E-07 | 0.500 | 2.0E-07 |
|  | Palm oil | 0.000 | 2.2E-07 | 0.500 | 2.2E-07 |
| **Fruits** | Palm oil + control | 0.010 | 2.1E-07 | 0.510 | 2.1E-07 |
|  | Palm oil + blueberries | 0.011 | 2.0E-04 | 0.511 | 2.0E-04 |
|  | Palm oil + blackberries | 0.015 | 8.8E-04 | 0.515 | 8.8E-04 |
|  | Palm oil + banana | 0.011 | 4.9E-05 | 0.511 | 4.9E-05 |

**Supplementary Table 3**: Caco-2 cell gene expression profiles of chylomicron-associated genes following treatment with digested palm oil, LPS, and digested BB, BK, or BN compared to control (Ctrl). Data are expressed as Log2 fold change (FC) relative to control. Statistical significance was defined as follows: ns (FDR > 0.05), * (FDR ≤ 0.05), ** (FDR ≤ 0.01), *** (FDR ≤ 0.001), **** (FDR ≤ 0.0001). Abbreviations: ABCA1 (ATP binding cassette subfamily A member 1), ACAT2 (Acetyl-CoA acetyltransferase, cytosolic), AGPAT2 (1-acyl-sn-glycerol-3-phosphate acyltransferase beta), APOA1 (Apolipoprotein A1), APOA4 (Apolipoprotein A4), APOB (Apolipoprotein B), DGAT2 (Diacylglycerol O-acyltransferase 2), FABP1 (Fatty acid binding protein 1), FABP2 (Fatty acid binding protein 2), GOT2 (Glutamic-oxaloacetic transaminase 2), MOGAT2 (Monoacylglycerol O-acyltransferase 2), MTTP (Microsomal triglyceride transfer protein), NPC1L1 (NPC1 like intracellular cholesterol transporter 1), PLA2G2A (Phospholipase A2 group IIA), PLPP1 (Phospholipid phosphatase 1), PLPP2 (Phospholipid phosphatase 2), and SLC27A1 (Solute carrier family 27 member 1).

| **Gene name** | **BB.vs.Ctrl.LPS** | | **BK.vs.Ctrl.LPS** | | **BN.vs.Ctrl.LPS** | |
| --- | --- | --- | --- | --- | --- | --- |
|  | **Log2FC** | **FDR** | **Log2FC** | **FDR** | **Log2FC** | **FDR** |
| *FABP2* | -1.888 | **** | -1.792 | **** | -1.365 | ** |
| *APOB* | -1.082 | *** | -0.842 | ** | -0.323 | ns |
| *ABCA1* | -0.831 | * | -0.931 | * | -0.403 | ns |
| *MTTP* | -0.825 | ** | -0.562 | ns | -0.291 | ns |
| *MOGAT2* | -0.563 | * | -0.280 | ns | -0.877 | * |
| *NPC1L1* | -0.507 | * | -0.305 | ns | -0.243 | ns |
| *ACAT2* | -0.442 | ** | -0.313 | * | -0.021 | ns |
| *FABP1* | -0.429 | * | -0.272 | ns | -0.306 | ns |
| *APOA4* | 0.145 | ns | -0.352 | ns | -0.518 | * |
| *PLA2G2A* | 0.233 | ns | 0.753 | ** | 0.284 | ns |
| *DGAT2* | 0.236 | * | 0.352 | ** | 0.044 | ns |
| *GOT2* | 0.323 | * | 0.223 | ns | -0.085 | ns |
| *AGPAT2* | 0.328 | * | 0.276 | ns | 0.082 | ns |
| *SLC27A1* | 0.342 | * | 0.255 | ns | 0.233 | ns |
| *PLPP1* | 0.363 | ** | 0.266 | ns | 0.222 | ns |
| *APOA1* | 0.415 | ** | 0.103 | ns | 0.112 | ns |
| *PLPP2* | 0.619 | ** | 0.659 | * | 0.229 | ns |

**Supplementary Table 4**: Caco-2 cell gene expression profiles of transcytosis-associated genes following treatment with digested palm oil, LPS, and digested BB, BK, or BN compared to control (Ctrl). Data are expressed as Log2 fold change (FC) relative to control. Statistical significance was defined as follows: ns (FDR > 0.05), * (FDR ≤ 0.05), ** (FDR ≤ 0.01), *** (FDR ≤ 0.001), **** (FDR ≤ 0.0001). Abbreviations: AP1G2 (Adaptor related protein complex 1 gamma 2 subunit),^[58]^ AP1S2 (Adaptor related protein complex 1 sigma 2 subunit),^[58]^ AP3M1 (Adaptor related protein complex 3 mu 1 subunit),^[58]^ CLTC (Clathrin heavy chain),^[94]^ CLINT1 (Clathrin interactor 1),^[94]^ DAB2 (DAB adaptor protein 2),^[49]^ EPS15 (Epidermal growth factor receptor pathway substrate 15),^[53]^ GOLGA1 (Golgin A1),^[62]^ GOLGA5 (Golgin A5),^[95]^ SNX2 (Sorting nexin 2),^[58]^ SNX4 (Sorting nexin 4),^[58]^ SNX6 (Sorting nexin 6),^[58]^ STAM2 (Signal transducing adaptor molecule 2),^[53]^ STON2 (Stonin 2),^[49]^ STX16 (Syntaxin 16),^[58]^ VPS26A (Vacuolar protein sorting-associated protein 26A),^[58]^ VPS36 (Vacuolar protein sorting 36 homolog),^[53]^ VPS37A (Vacuolar protein sorting 37 homolog A),^[53]^ VPS4B (Vacuolar protein sorting 4 homolog B)^[53]^, WASHC2A (WASH complex subunit 2A),^[59]^ WASHC2C (WASH complex subunit 2C).^[59]^

| **Gene name** | **BB.vs.Ctrl.LPS** | | **BK.vs.Ctrl.LPS** | | **BN.vs.Ctrl.LPS** | | **Localization** |
| --- | --- | --- | --- | --- | --- | --- | --- |
|  | **Log2FC** | **FDR** | **Log2FC** | **FDR** | **Log2FC** | **FDR** |  |
| *SNX4* | -0.982 | ** | -0.397 | ns | -0.159 | ns | Endosomes |
| *GOLGA5* | -0.926 | * | -0.567 | ns | 0.273 | ns | Golgi |
| *WASHC2C* | -0.878 | ** | -0.555 | ns | -0.218 | ns | Endosomes |
| *VPS36* | -0.861 | ** | -0.331 | ns | -0.181 | ns | Endosomes |
| *STAM2* | -0.815 | * | -0.433 | ns | -0.010 | ns | Endosomes |
| *SNX2* | -0.784 | * | -0.303 | ns | 0.024 | ns | Endosomes |
| *VPS4B* | -0.781 | * | -0.284 | ns | 0.059 | ns | Endosomes |
| *CLINT1* | -0.767 | ** | -0.298 | ns | 0.038 | ns | Endocytosis |
| *EPS15* | -0.766 | ** | -0.461 | ns | -0.082 | ns | Endosomes |
| *VPS26A* | -0.757 | * | -0.379 | ns | 0.223 | ns | Endosomes |
| *SNX6* | -0.716 | * | -0.261 | ns | 0.137 | ns | Endosomes |
| *STON2* | -0.683 | ** | -0.393 | ns | 0.018 | ns | Endocytosis |
| *DAB2* | -0.614 | *** | -0.590 | ** | -0.450 | * | Endocytosis |
| *AP1G2* | -0.608 | * | -0.587 | ns | -0.509 | ns | Golgi, endosomes |
| *AP1S2* | -0.587 | * | -0.305 | ns | 0.138 | ns | Golgi, endosomes |
| *GOLGA1* | -0.575 | ** | -0.227 | ns | -0.137 | ns | Golgi |
| *CLTC* | -0.573 | * | -0.163 | ns | 0.122 | ns | Endocytosis |
| *WASHC2A* | -0.572 | * | -0.310 | ns | 0.005 | ns | Endosomes |
| *VPS37A* | -0.570 | * | -0.255 | ns | -0.080 | ns | Endosomes |
| *STX16* | -0.373 | ** | -0.104 | ns | -0.143 | ns | Golgi |
| *AP3M1* | -0.360 | * | -0.087 | ns | -0.065 | ns | Endosomes |
